# The vast landscape of carbohydrate fermentation in prokaryotes

**DOI:** 10.1093/femsre/fuae016

**Published:** 2024-05-31

**Authors:** Timothy J Hackmann

**Affiliations:** Department of Animal Science, University of California, Davis, CA 95616, United States

**Keywords:** anaerobic metabolism, fermentation, genomics, metabolic pathways, microbial diversity, prokaryotes

## Abstract

Fermentation is a type of metabolism carried out by organisms in environments without oxygen. Despite being studied for over 185 years, the diversity and complexity of this metabolism are just now becoming clear. Our review starts with the definition of fermentation, which has evolved over the years and which we help further refine. We then examine the range of organisms that carry out fermentation and their traits. Over one-fourth of all prokaryotes are fermentative, use more than 40 substrates, and release more than 50 metabolic end products. These insights come from studies analyzing records of thousands of organisms. Next, our review examines the complexity of fermentation at the biochemical level. We map out pathways of glucose fermentation in unprecedented detail, covering over 120 biochemical reactions. We also review recent studies coupling genomics and enzymology to reveal new pathways and enzymes. Our review concludes with practical applications for agriculture, human health, and industry. All these areas depend on fermentation and could be improved through manipulating fermentative microbes and enzymes. We discuss potential approaches for manipulation, including genetic engineering, electrofermentation, probiotics, and enzyme inhibitors. We hope our review underscores the importance of fermentation research and stimulates the next 185 years of study.

## Teaser

Fermentation is a complex metabolism carried out by one-fourth of all prokaryotes and has several applications for agriculture, health, and industry.

## Introduction: Fermentation allows life to thrive without oxygen

Nearly 150 years ago, Louis Pasteur described fermentation as life without air (“la vie sans air”) (Pasteur 1876). During fermentation, organisms break down complex organic molecules (e.g. glucose) into simpler ones (e.g. lactate) (Hackmann and Zhang 2023). This process releases adenosine triphosphate (ATP) without requiring oxygen. Consequently, it is a major metabolic process for microbes in environments with little oxygen, such as the gut (Hillman et al. 2017, Mizrahi et al. 2021), sediments (Kessler et al. 2019), and anaerobic bioreactors (Khanal 2008).

Fermentation is important not only to microbes but also to human society. Pasteur studied fermentation in part because of its importance in the production of beer (Pasteur 1876). Fermentation products released by gut microbes are crucial to our health and are major energy sources for livestock (Bergman 1990, Koh et al. 2016). Fermentation products are also valuable as biofuels (Keasling et al. 2021) and other commodity chemicals (Lee et al. 2019, Varghese et al. 2022).

Despite the history and importance of fermentation, the diversity and complexity of this metabolism are just now becoming clear. Our review will explore this diversity and complexity by first examining which organisms carry out this metabolism. It will focus on recent studies that have analyzed written records of thousands of organisms and shown that over one-fourth of prokaryotes are fermentative. This review will also cover the complexity of fermentation at the biochemical level. It will map out pathways of glucose fermentation in unprecedented detail, showing the involvement of over 120 biochemical reactions. It will also cover recent studies revealing new pathways and enzymes, which underscore the hidden complexity of this metabolism. After summarizing these studies, this review will discuss applications of recent discoveries to human health, agriculture, and industry. It will begin, however, with defining fermentation—a surprisingly challenging exercise.

## What is fermentation?

While Pasteur defined fermentation as life without air, a number of more detailed definitions have emerged (Table 1). Details vary, but most define fermentation as catabolism where organic compounds are both electron donors and acceptors. Some definitions also allow inorganic compounds (e.g. protons) if they originate from metabolism. Some sources define fermentation loosely as any large-scale biological manufacturing process, but they will not be covered here.

**Table 1.** Several details definitions of fermentation have been proposed.

Definition	Source
“[B]iological processes that occur in the dark and that do not involve respiratory chains with oxygen or nitrate as electron acceptors.”	(Gottschalk 1986)
“[D]isproportionations of carbon compounds or redox reactions between carbon compounds.”	(Schink 1990)
“[A]n energy-yielding metabolic process (in the dark) in which an organic compound(s) serves as both an electron donor and an electron acceptor.”	(Meganathan et al. 2007)
“[A] process… [where] organic compounds are catabolized by strictly anaerobic or facultatively anaerobic bacteria by internally balanced oxidation–reduction reactions.… [T]he organic compound serves as both electron donor and acceptor, and adenosine triphosphate is synthesized by substrate level phosphorylation.”	(Müller 2008)
“[A] pathway in which NADH (or some other reduced electron acceptor that is generated by oxidation reactions in the pathway) is reoxidized by metabolites produced by the pathway… and ATP is produced by substrate-level phosphorylation.“	(White et al. 2012)
“[T]he donation of electrons onto terminal acceptors that are generated by the organism during metabolism, such as pyruvate (to produce lactate and/or opines), acetaldehyde (to produce ethanol), fumarate (to produce succinate), protons (to produce hydrogen), or acetyl-CoA (to produce fatty acids and their derivatives).”	(Müller et al. 2012)
“[A] form of anaerobic microbial growth using internally supplied electron acceptors and generating ATP mainly through substrate-level phosphorylation (SLP).“	(Kim and Gadd 2019)
“[A]naerobic catabolism in which an organic compound is both an electron donor and an electron acceptor and ATP is produced by substrate-level phosphorylation.”	(Madigan et al. 2021)
“[C]atabolism where organic compounds are both the electron donor and acceptor. Protons can be another electron acceptor, forming hydrogen (H_2_). H_2_ can be another electron donor but only if an organic electron donor (e.g. glucose) is also used.”	(Hackmann and Zhang 2023)

**Figure 1.**
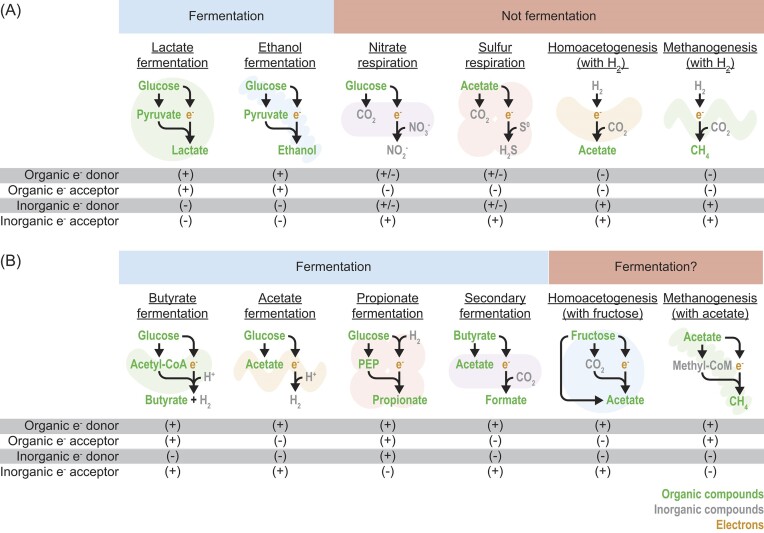
Fermentation can be distinguished from other types of anaerobic metabolism based on electron donors and acceptors involved. (A) Clear cases. (B) Edge cases. CoA = coenzyme A, CoM = coenzyme M, and PEP = phosphoenolpyruvate.

Any definition of fermentation should be tested to see how well it distinguishes fermentation from other types of metabolism. The simple definition (catabolism with organic compounds as electron donors and acceptors) works well in many cases (Fig. 1A). It correctly includes lactate and ethanol fermentation (Fig. 1A), the types of fermentation that appear most often in textbooks. It also correctly excludes nitrate respiration, sulfur respiration, homoacetogenesis, and methanogenesis (Fig. 1A). Like fermentation, these types of metabolism do not require oxygen and are carried out by many organisms in oxygen-deprived environments (Müller 2008). Moreover, it excludes anaerobic phototrophs, which use photosynthesis, not catabolism, to form ATP. The definition works well for these straightforward cases.

The simple definition does not work in all cases, however (Fig. 1B). It is problematic in cases where fermentation produces or consumes H_2_ (Fig. 1B). When acetate fermentation produces H_2_, the electron acceptors are protons (inorganic compounds). While it has been argued that protons come from metabolism (Müller et al. 2012), some come from water (Fig. S1) and may not originate from metabolism. During propionate fermentation, H_2_ can serve as a second electron donor (alongside organic compounds) [see Henderson (1980)]. The simple definition is also problematic for the secondary fermentation of butyrate, where CO_2_ can be used as an electron acceptor (Fig. 1B). These fermentations are not as well known as lactate or ethanol fermentations, but they are still carried out by many organisms (see below).

The simple definition of fermentation could be broadened to address some of these cases. It has been suggested the definition be broadened to include protons as an electron acceptor and H_2_ as a second electron donor (Hackmann and Zhang 2023) (Table 1). The definition could likewise be broadened to include CO_2_ as an electron acceptor. However, if this is done, then homoacetogenesis, not normally classified as a fermentation, would become one when it uses fructose or other organic compounds (Fig. 1B). We recommend using the broader definition, and leaving it to the investigator to decide how to handle edge cases. There are additional edge cases, such as methanogenesis with acetate (Fig. 1B), that are difficult to resolve with any definition.

Beyond defining what electron donors and acceptors are used, many definitions also specify how fermentation forms ATP (Table 1). Often, definitions specify that fermentation forms ATP through substrate-level phosphorylation, rather than through an electron transport chain and ATP synthase. While this definition made sense long ago, electron transport chains have been known in fermentative organisms for over 60 years (White et al. 1962). In one organism recently studied, the electron transport chain and ATP synthase were found to form up to 1/3 of the total ATP (Hackmann and Firkins 2015, Schoelmerich et al. 2020). Thus, we recommend that fermentation not be defined according to how ATP is formed.

In sum, most cases of fermentation involve catabolism with organic compounds acting as electron donors and acceptors. For the purposes of this review, fermentation can also use protons or CO_2_ as electron acceptors, and it can use H_2_ as an electron donor alongside organic compounds. While methanogenesis and homoacetogenesis meet one or more criteria for fermentation, they are not classified as types of fermentation in this review.

## Fermentative organisms are diverse

### Recent studies reveal unprecedented diversity

Fermentation was first described in yeast by three separate investigators over 185 years ago (Kützing 1837, Schwann 1837, Cagniard-Latour 1838) (Fig. S2). Since that time, it has been described in a range of organisms. The first attempts to classify fermentative organisms and describe their traits appeared in the early 1900s (Orla-Jensen 1909, Bergey et al. 1923) (Fig. S2). While most species and genera they described are no longer recognized, a few (such as *Lactobacillus* and *Propionibacterium*) are still valid. These early works evolved into reference books, such as the modern edition of *Bergey's Manual* (Whitman 2024), that have thousands of descriptions. These descriptions have a wealth of information, but they are made up of long and unstructured text that is difficult to summarize. Consequently, it has been challenging to understand the full diversity and abundance of fermentative organisms.

Recent studies have extracted information on fermentation from written descriptions, helping reveal the full diversity of this metabolism. The first study to do so, relying largely on *Bergey's Manual*, found that fermentation is carried out by 162 different genera of prokaryotes (Louca et al. 2016) (Fig. S2). This study used this knowledge to predict which organisms in the ocean microbiome were fermentative, finding that they were widely distributed. Given it was a large study covering many types of metabolism, information on fermentation specifically was more limited. However, it provided early evidence that fermentative microbes were diverse and widespread.

**Figure 2.**
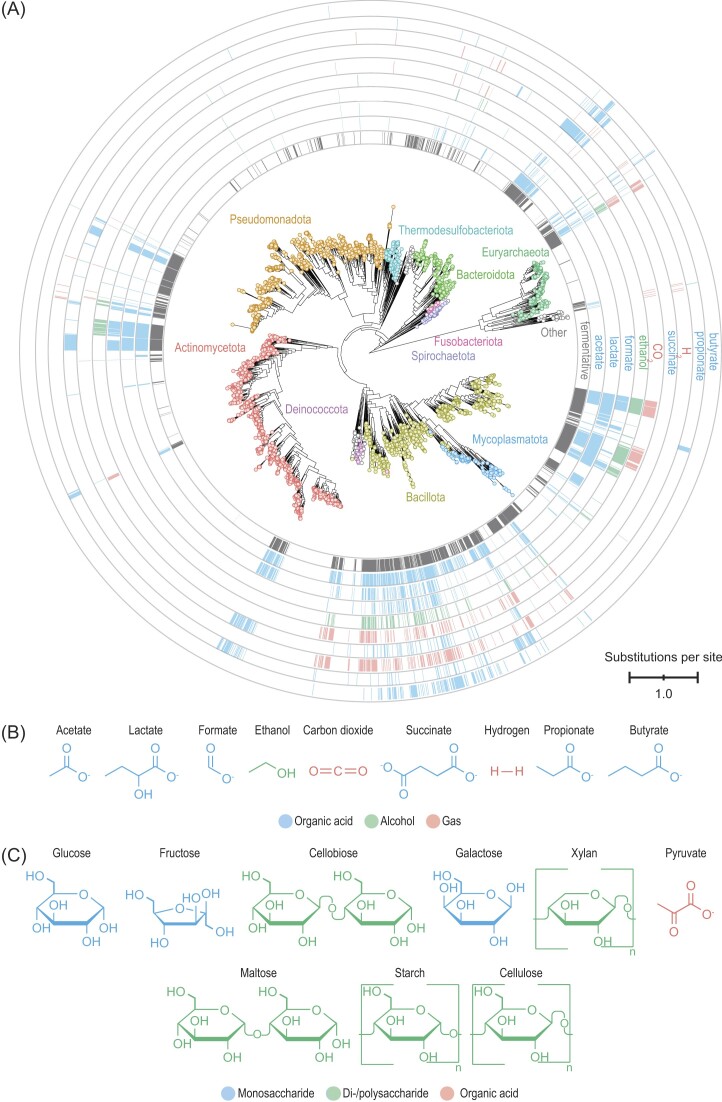
Fermentative prokaryotes are diverse, produce a range of end products, and use a range of substrates. (A) Phylogenetic tree of prokaryotes, highlighting those that are fermentative and their end products. (B) The nine most common end products of fermentation. (C) The nine most common substrates of fermentation. The figure is drawn from data in Hackmann and Zhang (2023).

More recent studies have focused on fermentation exclusively and revealed more insights (Fig. 2). These studies examined descriptions of 8 300 prokaryotes, using both *Bergey's Manual* and the primary literature, and they found over 1/4 of these organisms carry out fermentation (Hackmann and Zhang 2021, 2023). The definition of fermentation used in these studies is shown in Table 1. They showed fermentative organisms are found across the tree of life (Fig. 2A), though they are more concentrated in some phyla than others. Also evident is that fermentation forms a range of metabolic end products (Fig. 2A and B). Acetate and lactate are the most commonly formed products, but 55 end products are formed in all (Hackmann and Zhang 2023). Most organisms form multiple end products, and nearly 300 combinations are observed (Hackmann and Zhang 2023). Further, fermentation uses a range of substrates (Fig. 2C). Carbohydrates and pyruvate are those most commonly used (Fig. 2C), but 46 chemically-defined substrates have been reported in total (Hackmann and Zhang 2023). These findings show a vast diversity and complexity of fermentation. Given that not all organisms, end products, and substrates have been described, the true diversity of fermentation is even greater than these studies suggest.

### More work is still needed to uncover all diversity

The ability to study this range of organisms owes largely to advances in data science. Extracting data from descriptions of books and the primary literature is tedious, but it has now been partly automated computationally (Hackmann and Zhang 2021). Advances in computational techniques, including large language models, should make it possible to extract even more data. Large language models, such as the GPT-4 model underlying ChatGPT (OpenAI 2023), may soon achieve human-level accuracy in data extraction.

Another opportunity for this field is to investigate eukaryotic and uncultured microbes. Eukaryotes, from microbes to animals, can carry out fermentation (Müller et al. 2012), but comparatively little information about them is available. Uncultured microbes make up the vast majority of microbial diversity (Nayfach et al. 2021), but given the inability to grow them in the lab, information is again limited. There have been large-scale projects to culture organisms, including from fermentative environments (Lagier et al. 2016, Lagkouvardos et al. 2016, Wylensek et al. 2020), and these efforts must continue. With these efforts, we will work towards the goal of revealing the full diversity of fermentative organisms.

## Diversity of fermentation extends to the biochemical level

### The study of fermentation biochemistry has a rich history

Fermentation is carried out by a range of organisms, and this diversity extends to the biochemical level. Attempts to uncover that diversity trace back to Eduard Buchner's discovery of enzymes nearly 130 years ago (Buchner 1897) (Fig. S2). Buchner proposed that fermentation in yeast was carried out by a single enzyme (zymase). However, reality turned out to be more complex, and several decades were spent purifying the responsible enzymes. The work was painstaking, and one paper purified seven separate enzymes to outline one pathway of propionate formation (Allen et al. 1964). By 1986, major pathways revealed with this approach were summarized in a landmark textbook by Gerhard Gottschalk (1986). These same pathways would be rendered, almost unchanged, in textbooks and other resources for decades to come (Meganathan et al. 2007, Müller 2008, White et al. 2012, Kim and Gadd 2019).

While our understanding of fermentation appeared mostly complete decades ago, recent studies have pointed to several gaps. In many cases, these gaps have been identified and filled using genomics, an approach that has transformed the field of fermentation biochemistry. This review will sum up all progress—from early work to recent discoveries—by first focusing on the fermentation of glucose.

### Fermentation of glucose follows several pathways

**Figure 3.**
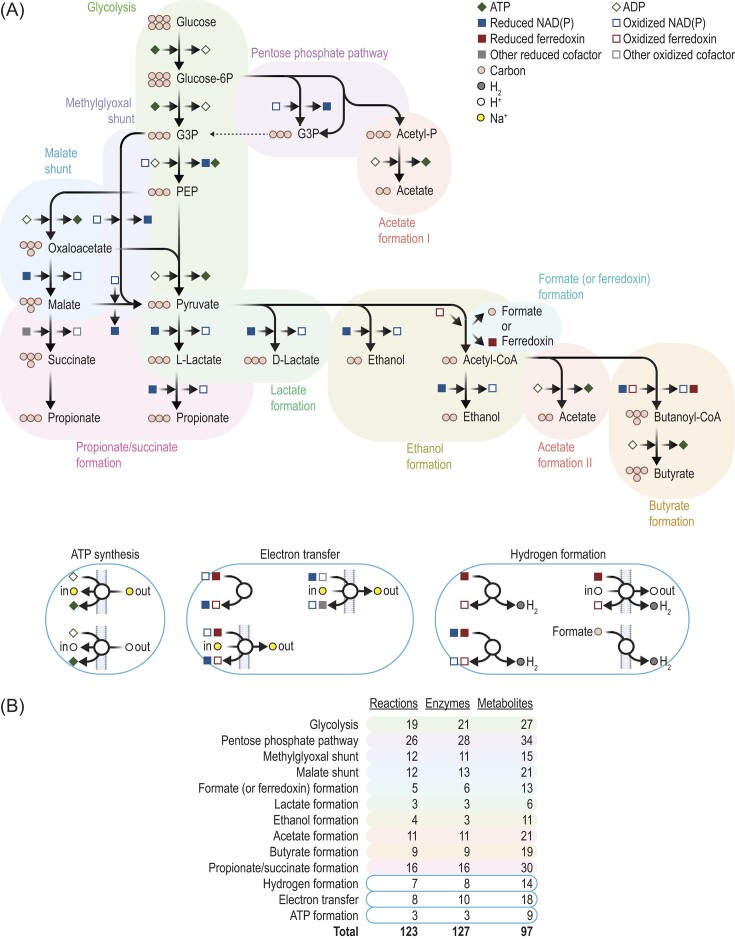
The biochemical pathways for the fermentation of glucose can be summarized in the outline. (A) Pathways. (B) A number of biochemical reactions, enzymes, and metabolites. Panel A drawn from Hackmann et al. (2017), Hackmann and Zhang (2023), and references in text. A full version is available in poster format as in Fig. S3 and in table format in Table S1. Reduced ferredoxin refers to two reduced iron–sulfur clusters. CoA = coenzyme A, G3P = glyceraldehyde-3P, PEP = phosphoenolpyruvate, and -P = phosphate.

Given that glucose is the most common substrate of fermentation (Fig. 2C), the biochemistry of glucose fermentation has been studied in great detail. The pathways that have emerged are outlined in Fig. 3 and presented in full in Fig. S3.

When pathways are viewed in outline (Fig. 3A), fermentation is simple. Glucose enters either through glycolysis or the pentose phosphate pathway and is converted to pyruvate. From pyruvate, pathways branch out to form a number of end products (e.g. lactate). At several points, redox cofactors (NAD and ferredoxin) accept electrons and become reduced. At later points, these cofactors donate electrons and become oxidized. ATP is also formed at several points in the pathway, either in fermentation reactions directly (by substrate-level phosphorylation) or by ATP synthase. This textbook picture of fermentation suggests a low level of complexity to this metabolism.

When fermentation reactions are viewed in full (Fig. S3), the complexity of this metabolism emerges. Across organisms, fermentation involves a total of 123 biochemical reactions, 127 enzymes, and 97 metabolites (Figs. 3B, S3, Table S1). Additionally, multiple pathways can be responsible for forming the same product. For forming acetate from its immediate precursor (pyruvate or acetyl-CoA), six separate pathways have been found (Fig. S3). The process of electron transfer is also complex, and there are a number of enzymes just for interconverting reduced cofactors (Fig. 3B, S3, Table S1).

### Recent studies shed insight into the fermentation of glucose

**Figure 4.**
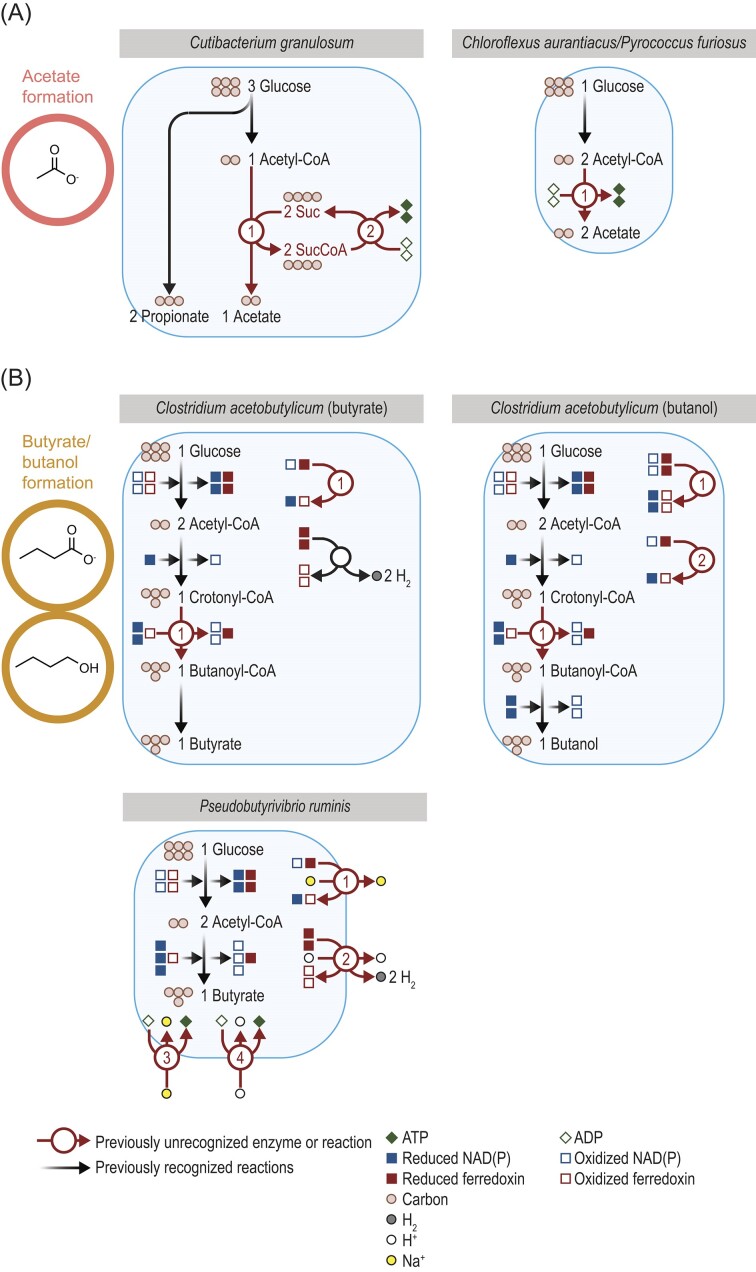
Several organisms use previously unrecognized enzymes in the fermentation of glucose to (A) acetate or (B) butyrate or butanol. Stoichiometry of ATP formation for acetate is shown in (A) but not (B). Stoichiometry of redox cofactors is shown in full in (B) but not (A). Stoichiometry of ions has been simplified. Reduced ferredoxin refers to two reduced iron–sulfur clusters. Enzymes for *Chloroflexus aurantiacus* and *Pyrococcus furiosus*: 1, acetate—CoA ligase (ADP-forming) (EC 6.2.1.13). Enzymes for *Cutibacterium granulosum*: 1, succinyl-CoA:acetate CoA-transferase (EC 2.8.3.18); and 2, succinate—CoA ligase (ADP forming) (EC 6.2.1.5). Enzymes for *Clostridium acetobutylicum*: 1, ferredoxin—NAD^+^ reductase (EC 1.18.1.3) or butyryl-CoA dehydrogenase; EC 1.3.8.1; and 2, ferredoxin—NADP^+^ reductase (EC 1.18.1.2). Enzymes for *Pseudobutyrivibrio ruminis*: 1, ferredoxin—NAD^+^ oxidoreductase (Na^+^-transporting) (Rnf) (EC 7.2.1.2); 2, energy-converting hydrogenase (Ech) (EC 7.1.1.-); 3, Na^+^-transporting two-sector ATPase (EC 7.2.2.1); and 4, H^+^-transporting two-sector ATPase (EC 7.1.2.2). CoA = coenzyme A, G3P = G3P = glyceraldehyde-3P, PEP = phosphoenolpyruvate, -P = phosphate, suc = succinate, and sucCoA = succinyl-CoA.

**Figure 5.**
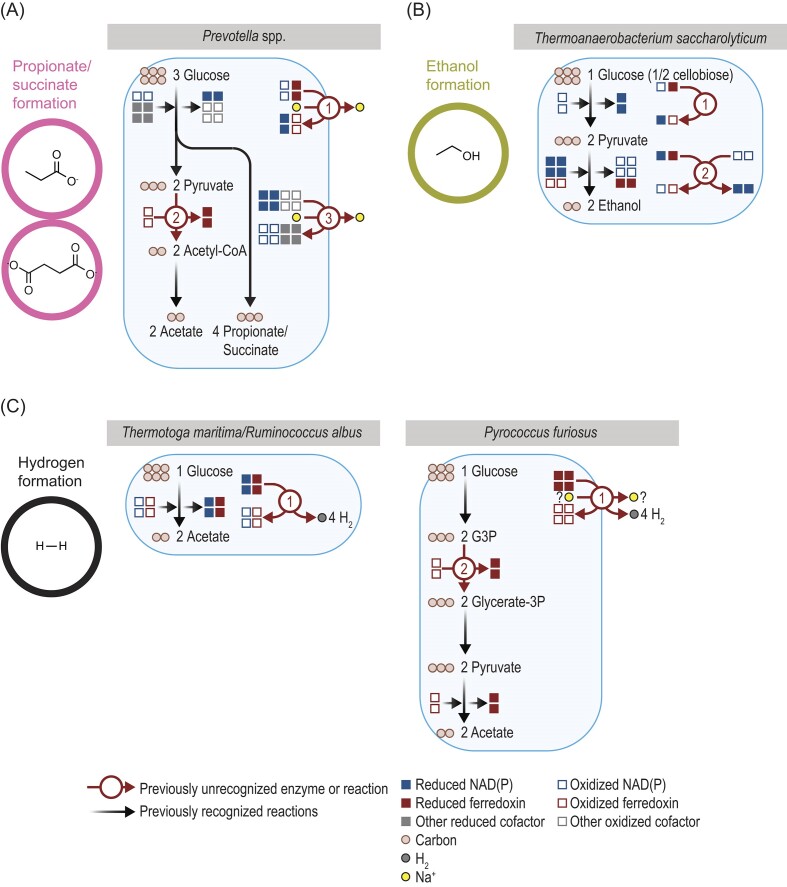
Several organisms use previously unrecognized enzymes in the fermentation of glucose to (A) propionate or succinate, (B) ethanol, or (C) H_2_. Stoichiometry of ATP formation is not shown. Stoichiometry of ions has been simplified. Reduced ferredoxin refers to two reduced iron–sulfur clusters. Enzymes for *Prevotella* spp.: 1, ferredoxin—NAD^+^ oxidoreductase (Na^+^-transporting) (Rnf) (EC 7.2.1.2); and 2, pyruvate:ferredoxin oxidoreductase (EC 1.2.7.1). Enzymes for *Thermoanaerobacterium saccharolyticum*: 1, ferredoxin—NAD^+^ reductase (EC 1.18.1.3); and 2, NAD(P)^+^ transhydrogenase (ferredoxin) (Nfn) (EC 1.6.1.4). Enzymes for *Thermotoga maritima* and *Ruminococcus albus*: 1, hydrogenase (NAD^+^, ferredoxin) (EC 1.12.1.4). Enzymes for *Pyrococcus furiosus*: 1, membrane-bound hydrogenase (Mbh) (EC 7.2.1.-); and 2, glyceraldehyde-3-phosphate dehydrogenase (ferredoxin) (EC 1.2.7.6). CoA = coenzyme A, G3P = G3P = glyceraldehyde-3P, PEP = phosphoenolpyruvate, -P = phosphate, suc = succinate, and sucCoA = succinyl-CoA.

Examining discoveries from recent studies helps illustrate the complexity of glucose fermentation. These studies have discovered new pathways and enzymes for forming acetate, butyrate, butanol, propionate, ethanol, and H_2_ (Figs. 4, 5). For acetate, two new pathways have been discovered. In *Cutibacterium granulosum* (Fig. 4A), acetate was found to be formed from acetyl-CoA with two unrecognized enzymes (Zhang et al. 2021). These enzymes are succinyl-CoA:acetate CoA-transferase (EC 2.8.3.18) and succinyl-CoA synthetase [adenosine diphosphate (ADP) forming] (EC 6.2.1.5). They were found after searching the genome revealed no alternatives. Biochemical experiments confirmed these two enzymes are active and form a functional pathway for forming acetate. The pathway they form is analogous to one described in eukaryotes (Lindmark 1976), but the enzymes used by *C. granulosum* are bacterial in origin. The enzymes and pathway have been subsequently detected in uncultured bacteria (Kumar et al. 2022). In *Chloroflexus aurantiacus* (Fig. 4A), acetate was found to be formed from acetyl-CoA by one enzyme (acetate—CoA ligase [ADP-forming]; EC 6.2.1.13) (Schmidt and Schonheit 2013). Other enzymes for forming acetate were absent in the genome, and the current enzyme was purified to homogeneity to confirm its existence. It is similar to an enzyme from *Pyrococcus furiosus*, an archaeon, which was found by traditional purification procedures (Schäfer and Schönheit 1991, Musfeldt et al. 1999). Pathways for forming acetate in bacteria have been studied since 1940 (Lipmann 1939), and these recent discoveries underscore the amount of hidden diversity.

Recent studies shed insight into the fermentation of glucose to butyrate or butanol. In *Clostridium acetobuylicum* (Fig. 4B), two enzymes were found important in the balance of redox cofactors during the formation of butyrate and butanol (Yoo et al. 2015, Foulquier et al. 2022). One enzyme (ferredoxin—NAD^+^ reductase; EC 1.18.1.3) transfers electrons from reduced ferredoxin to oxidized NAD. A second enzyme (ferredoxin—NAD^+^ reductase; EC 1.18.1.3) carries out a similar transfer, but to oxidized NADP. These enzymes ensure that fermentation is balanced and there is no build-up of reduced ferredoxin or oxidized NAD(P). Both enzymes were found by traditional purification procedures after searching the genome revealed no alternatives. The first enzyme turned out to be a previously known enzyme (butyryl-CoA dehydrogenase; EC 1.3.8.1), and the new role (EC 1.18.1.3) was discovered after this purification. In *Pseudobutyrivibrio ruminis* (Fig. 4B), two enzymes were found to be an important balance of redox cofactors during the formation of butyrate (Schoelmerich et al. 2020). Both enzymes, Rnf (ferredoxin—NAD^+^ oxidoreductase [Na^+^-transporting]; EC 7.2.1.2) and Ech (energy-converting hydrogenase; EC 7.1.1.-) transfer electrons away from reduced ferredoxin to stop build-up of this cofactor. Further, these enzymes pump out sodium (Kuhns et al. 2020) and hydrogen ions (Katsyv and Müller 2022). These ions in turn drive ATP formation by two different ATP synthases (EC 7.2.2.1, EC 7.1.2.2), and so these enzymes also play an important role in energy conservation. This is the first example of an organism that forms ATP with two ions and two ATPases, and it shows the diversity of bacterial metabolism.

Recent studies found previously unrecognized enzymes in forming propionate and succinate. In *Prevotella* spp. (Fig. 5A), three unrecognized enzymes have been found in two studies (Schleicher et al. 2021, Zhang et al. 2023). The first study (Zhang et al. 2023) found that Rnf is present, and as in *P. ruminis*, this enzyme transfers electrons away from reduced ferredoxin. This is important because a second enzyme was found that forms reduced ferredoxin. This second enzyme, pyruvate:ferredoxin oxidoreductase (EC 1.2.7.1), is central to the metabolism of many fermentative organisms, and without Rnf, it would quickly make fermentation unbalanced. Historically, this problem was overlooked because pyruvate:ferredoxin oxidoreductase was not recognized as part of this pathway (succinate pathway) (Allen et al. 1964). It is likely that these two enzymes are also used by *Anaerotignum neopropionicum* (Moreira et al. 2021), which forms propionate with an unrelated pathway (acrylate pathway). The second study (Schleicher et al. 2021) found a third previously unrecognized enzyme (Nqr; EC 7.2.1.1). Enzymes similar to Nqr have been found in this pathway, but Nqr is unique in pumping out sodium. This adds to the sodium pumped out by Rnf and allows the organisms to conserve additional energy.

Recent studies have found unrecognized enzymes in one organism forming ethanol. In *Thermoanaerobacterium saccharolyticum* (Fig. 5B), two enzymes were found important in maximizing the yield of ethanol over other products (e.g. acetate) (Lo et al. 2015, Tian et al. 2016). The first enzyme (ferredoxin—NAD^+^ reductase; EC 1.18.1.3) transfers electrons from reduced ferredoxin to oxidized NAD. This is the same reaction carried out by the enzyme in *Clostridium acetobuylicum* (Fig. 4B), though the amino acid sequences of the enzymes are not related. The second enzyme, Nfn (NAD[P]^+^ transhydrogenase [ferredoxin]; EC 1.6.1.4), transfers electrons from reduced ferredoxin and NAD to oxidized NADP. Together, these enzymes balance redox cofactors and theoretically allow fermentation to form only ethanol (though in practice some acetate, lactate, and H_2_ are still formed). If genes for both are deleted, the ethanol yield decreases (Tian et al. 2016).

One final set of studies showed new enzymes for forming H_2_. In *Thermotoga maritima* (Fig. 5C), H_2_ was found to be formed by hydrogenase (NAD^+^, ferredoxin) (EC 1.12.1.4) (Schut and Adams 2009). This hydrogenase uses both reduced NAD and reduced ferredoxin, making it the first hydrogenase known to use more than one type of redox cofactor. It is crucial for balancing fermentation, which produces both reduced NAD and reduced ferredoxin. This enzyme was later found to play the same role in *Ruminococcus albus* (Zheng et al. 2014). This enzyme is otherwise well known because it uses a type of energy coupling known as electron confurcation (see below). In *Pyrococcus furiosus* (Fig. 5C), it was found that H_2_ was formed by a membrane-bound hydrogenase (Mbh, EC 7.2.1.-) (Sapra et al. 2003). This hydrogenase partners with a second enzyme that produces reduced ferredoxin (glyceraldehyde-3-phosphate dehydrogenase [ferredoxin]; EC 1.2.7.6). The hydrogenase not only forms H_2_ but also likely pumps sodium (Yu et al. 2018) or hydrogen ions (Sapra et al. 2003), which could drive ATP formation. A related hydrogenase, Ech, was later found to play a similar role in *Pseudobutyrivibrio ruminis* (Fig. 4B).

**Figure 6.**
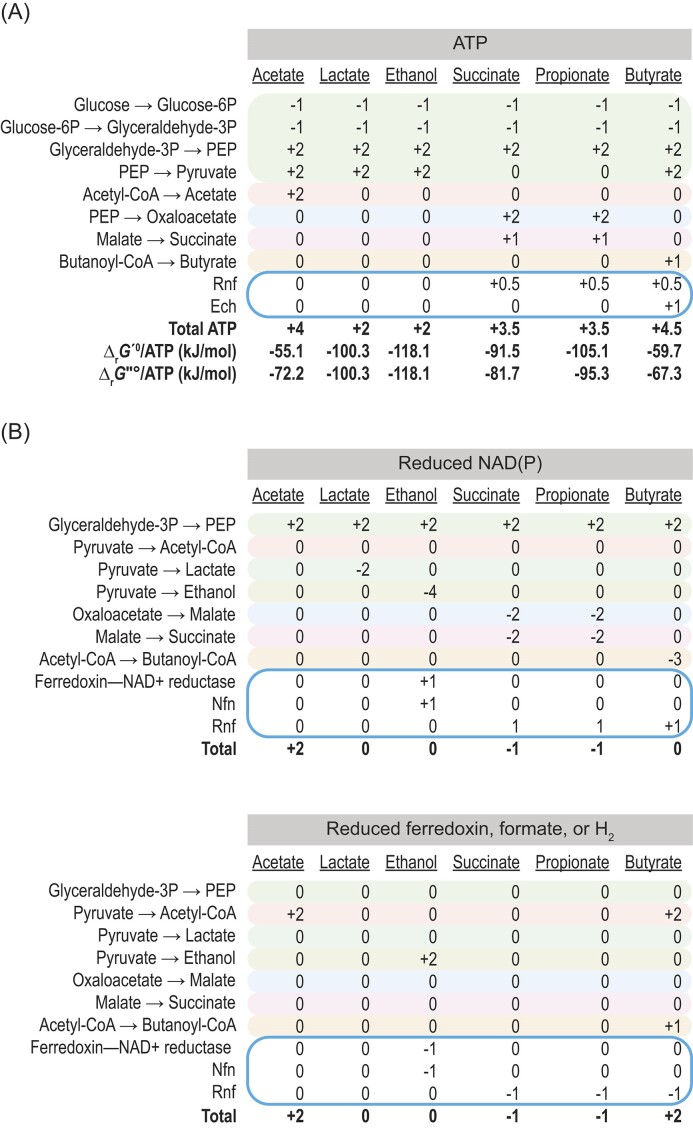
Summary of ATP and reduced cofactors formed in fermentation of glucose to different end products. (A) ATP. (B) Reduced cofactors. Formate and H_2_ are treated as equivalent to reduced ferredoxin. Values are mol/mol glucose unless otherwise specified. Values for acetate are based on *Thermotoga maritima* and *Ruminococcus albus*; values for ethanol are based on *Thermoanaerobacterium saccharolyticum*; values for succinate and propionate are based on *Prevotella* spp.; and values of butyrate are based on *Pseudobutyrivibrio ruminis*. See Figs. 4,  5, S3 for more details. Values for lactate are those typical for lactic acid (Gottschalk 1986). Most organisms form multiple products, requiring values pertaining to the entire fermentation to be split arbitrarily. Transport of glucose and fermentation products across the cell membrane is not represented, though this can affect the yield of ATP. Standard transformed Gibbs energy of reaction (∆_r_*G*′°) was calculated following Alberty (2003) and Hackmann et al. (2013) with *T* = 298.15 K, *I* = 0.25, and pH = 7. Reaction stoiochiometry was taken from Hackmann et al. (2013), except for succinate, which was taken as 1 glucose(aq) + 2 H_2_(g) + 2 CO_2_(g) = 2 succinate(aq) + 2 H_2_O(l). Concentrations were 1 *M* for aqueous reactants and 1 bar for gaseous reactants. The standard further transformed Gibbs energy of reaction (∆_r_*G*″°) was calculated similarly but with H_2_(g) = 1∙10^−3^ bar, the highest value observed for most environments (Thauer et al. 1977).

Several previously unrecognized enzymes help form ATP or balance redox cofactors. Their impact becomes clear when summing up ATP and reduced redox cofactors from fermentation (Fig. 6). Rnf and Ech, e.g. increase the yield of ATP by up to 50% when butyrate is formed. Consequently, they decrease the Gibbs energy (∆_r_*G*) per ATP and bring it near −60 kJ/mol, the minimum energy observed for most types of metabolism (Schink 1990, 1997). Butyrate fermentation was once considered energetically inefficient (Thauer et al. 1977, Kohn and Boston 2000), but it is now clear it can be just as efficient in forming ATP as other types of fermentation.

In summary, recent work underscores the complexity of the fermentation at the biochemical level. It shows that even for fermentation of glucose, a well-studied process, there are many enzymes and pathways that had been previously unrecognized. Many of these enzymes are involved in the balance of redox cofactors, and no doubt there are many to still be discovered.

### Recent studies shed insight into the fermentation of amino acids and other substrates

While most recent studies have focused on the fermentation of glucose, there have been advances in understanding other fermentation pathways. We cover major advances since the landmark textbook by Gottschalk (1986).

**Figure 7.**
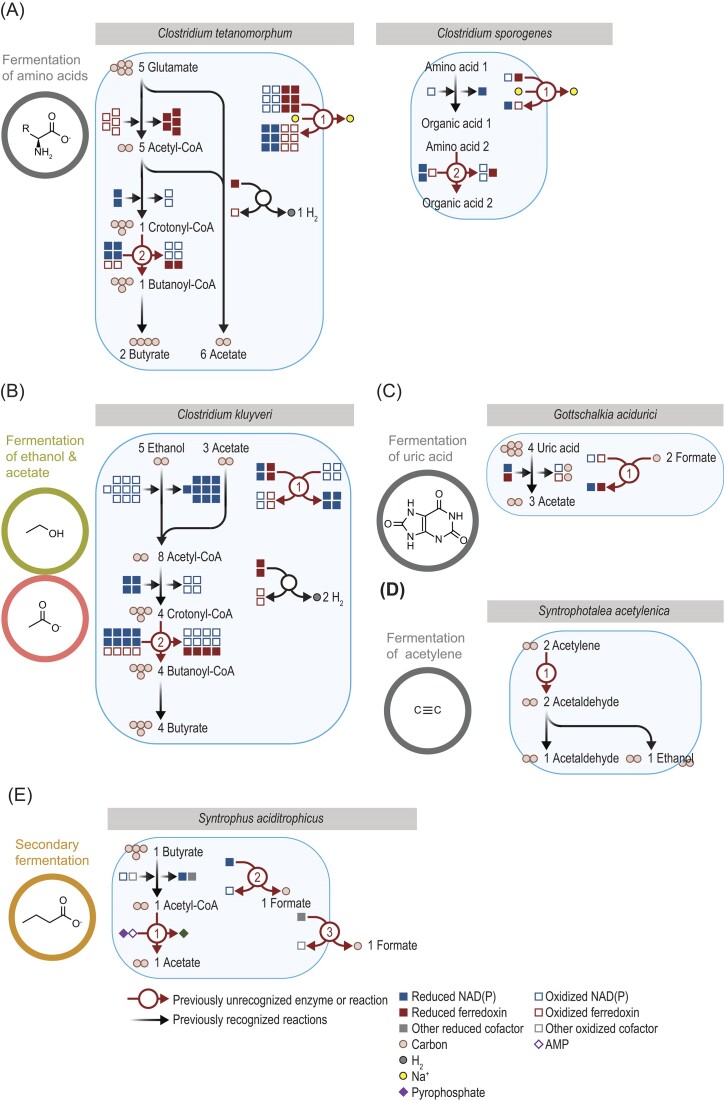
Several organisms use previously unrecognized enzymes in the fermentation of amino acids or other substrates. Organisms are arranged by those fermenting (A) amino acids, (B) ethanol and acetate, (C) uric acid, (D) acetylene, or (E) butyrate. Stoichiometry of ATP formation is not shown. Stoichiometry of redox cofactors not shown in (D). Stoichiometry of ions has been simplified. Reduced ferredoxin refers to two reduced iron–sulfur clusters. Enzymes for *Clostridium tetanomorphum*: 1, ferredoxin—NAD^+^ oxidoreductase (Na^+^-transporting) (Rnf) (EC 7.2.1.2); and 2, butyryl-CoA dehydrogenase (EC 1.3.8.1). Enzymes for *Clostridium sporogenes*: 1, ferredoxin—NAD^+^ oxidoreductase (Na^+^-transporting) (Rnf) (EC 7.2.1.2); and 2, acyl-CoA dehydrogenase, such as 3-(aryl)acrylate reductase (EC 1.3.8.15) or butyryl-CoA dehydrogenase (EC 1.3.8.1). Enzymes for *Clostridium kluyveri*: 1, NAD(P)^+^ transhydrogenase (ferredoxin) (Nfn) (EC 1.6.1.4); and 2, butyryl-CoA dehydrogenase (EC 1.3.8.1). Enzymes for *Gottschalkia acidurici*: 1. formate dehydrogenase (NAD^+^, ferredoxin) (EC 1.17.1.11). Enzyme for *Syntrophotalea acetylenica*: 1, acetylene hydratase (EC 4.2.1.112). Enzymes for *Syntrophus aciditrophicus*: 1., acetate—CoA ligase (EC 6.2.1.1); 2, electron-transferring flavoprotein:methylmenaquinone oxidoreductase (EMO) (EC 1.5.5.1) and formate dehydrogenase-N (EC 1.17.5.3); and 3, formate dehydrogenase (EC 1.17.1.9). CoA = coenzyme A.

There have been several advances in understanding the fermentation of amino acids. In *Clostridium tetanomorphum* (Fig. 7A), Rnf and a second enzyme were found to balance redox cofactors during the fermentation of glutamate (Jayamani 2008, Buckel and Thauer 2013). This was the first fermentation in which Rnf was reported, and it set the stage for understanding its role in other pathways (see Figs. 4B,  5A). The second enzyme, butyryl-CoA dehydrogenase (EC 1.3.8.1), reduces crotonyl-CoA to butyryl-CoA and forms both reduced ferredoxin and oxidized NAD in the process. This enzyme was previously known, but it was previously thought to form oxidized NAD only. This discovery also set the stage for understanding its role in other pathways (see Figs. 4B,  7B). In *Clostridium sporogenes* (Fig. 7A), a similar role for these two enzymes exists (Liu et al. 2022). Historically, it was believed this organism ferments amino acids by simply transferring electrons from one amino acid to another (Stickland 1934). However, electron transfer is more complex, and both butyryl-CoA dehydrogenase and Rnf are involved. Depending on the amino acid fermented, butyryl-CoA dehydrogenase is replaced with 3-(aryl)acrylate reductase (EC 1.3.8.15) or an analogous acyl-CoA dehydrogenase. *Acidaminococcus fermentans* (Buckel and Thauer 2013) and *Clostridium difficile* (Neumann-Schaal et al. 2019) are other organisms that ferment amino acids and have genes for Rnf and acyl-CoA dehydrogenases. This set of enzymes thus appears prevalent in this type of metabolism.

One of the most unusual and longest-studied fermentations is in *Clostridium kluyveri*. In this organism, both ethanol and acetate are simultaneously fermented (Fig. 7B). Two enzymes have been discovered as crucial in balancing redox cofactors in this fermentation (Fig. 7B) (Li et al. 2008, Wang et al. 2010). One enzyme, butyryl-CoA dehydrogenase (EC 1.3.8.1), serves the same role as in *C. tetanomorphum* (Fig. 7A), where it was characterized around the same time. The second enzyme, Nfn, serves the same role as in *T. saccharolyticum* (Fig. 5B), and it was characterized first in C. *kluyveri*. Together, these enzymes balance fermentation (Buckel and Thauer 2013). It is possible that redox cofactors are balanced by Rnf, also, (Buckel and Thauer 2013), given genes for it have been found in the genome (Seedorf et al. 2008). However, the catalytic activity of Rnf has not been demonstrated experimentally.

*Gottschalkia acidurici* ferments uric acid, and one study shed insight into this process (Fig. 7C). This study (Wang et al. 2013) found the enzyme formate dehydrogenase (NAD^+^, ferredoxin) (EC 1.17.1.11) forms both reduced ferredoxin and reduced NAD. This enzyme was previously known, but it was unclear which reduced cofactors it formed. By forming both of these reduced cofactors, the enzyme balances other reactions forming oxidized cofactors.

Acetylene is an organic compound with a carbon–carbon triple bond, making it an unlikely substrate for fermentation. However, the organism *Syntrophotalea acetylenica* can indeed ferment this compound to acetate and ethanol (Fig. 7D) (Schink 1985), and one recent study gives insight into this process. This study (Seiffert et al. 2007) showed the carbon–carbon triple bond is broken by the enzyme acetylene hydratase (EC 4.2.1.112), and it accomplishes this by using water and tungsten as a cofactor. Redox cofactors are not used at this step, though both NAD and ferredoxin are used in later steps of the pathway (Schink 1985).

Two recent studies have studied *Syntrophus aciditrophicus* to shed insight into secondary fermentation (Fig. 7E). Secondary fermentation involves fermenting butyrate or other end products from other microbes, forming even simpler molecules. One study (James et al. 2016) showed this organism forms acetate from acetyl-CoA with a single enzyme (acetate—CoA ligase; EC 6.2.1.1). This enzyme is unique because it forms ATP from adenosine monophosphate (AMP) and pyrophosphate, not ADP and inorganic phosphate. By using this enzyme, the organism can conserve an extra 0.75 ATP (assuming 1 ATP is saved by not synthesizing ADP from AMP and 0.25 ATP is spent on synthesizing pyrophosphate from inorganic phosphate; see Fig. S3). This enzyme is well known for its role in anabolism, and this is the first case where it was shown to have a catabolic function. A second study (Agne et al. 2021b) showed how this organism balances redox cofactors. This study revealed how electrons are transferred from reduced cofactors to CO_2_, forming formate in the process. While some enzymes were known from other pathways, one enzyme, EMO (electron-transferring flavoprotein:methylmenaquinone oxidoreductase; EC 1.5.5.1), was unknown and newly characterized in this study. The enzyme EMO has since been found involved in another pathway of secondary fermentation (Agne et al. 2021a), and it could be key to manipulating this process.

### Some pathways still have gaps remaining

While recent studies have found and filled many apparent gaps in fermentation pathways, others still remain. One gap that still remains is in the bacterium *Butyrivibrio proteoclasticus*, which lacks enolase, a key enzyme of glycolysis (Kelly et al. 2010). It is still not clear what enzyme or enzymes may substitute; the methylglyoxal shunt was proposed as an alternative (Kelly et al. 2010), but not all needed enzymes are encoded by the genome (Hackmann et al. 2017). The problem is heightened by the discovery of more bacteria lacking this seemingly essential enzyme (Hackmann et al. 2017, Seshadri et al. 2018). While genomics can highlight gaps, more classic biochemical experiments are needed to fill them.

Another outstanding gap is in the pathway for forming propionate. While the pathway in *Prevotella* spp. is now clear (Fig. 5A), it is still unclear in propionibacteria, where the pathway was first delineated (Allen et al. 1964). It is now known that propionibacteria form reduced ferredoxin via pyruvate:ferredoxin oxidoreductase; knocking out this enzyme results in poor growth (McCubbin et al. 2020). However, it is unclear which enzyme transfers electrons from reduced ferredoxin to oxidized NAD; propionibacteria do not have genes for Rnf. While some possible enzymes have been proposed (McCubbin et al. 2020, Dank et al. 2021), they need to be verified with biochemical experiments.

### Reconstructing fermentation pathways from genomes has promises and pitfalls

**Figure 8.**
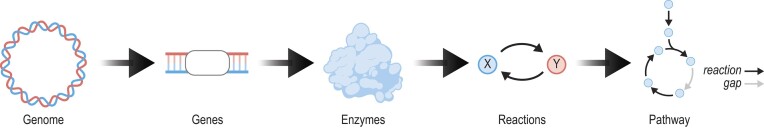
An organism's metabolic pathways can be reconstructed from its genome in several steps.

A common theme of recent studies is they find gaps in fermentation pathways by using genomics. How does this process work? It is conceptually straightforward (Fig. 8). The organism's genome is first searched for genes for enzymes catalyzing biochemical reactions, then the reactions are assembled into pathways. The predicted (reconstructed) pathways are examined for gaps, where a reaction is apparently missing. The gaps are then filled by searching for alternate enzymes (ones not previously recognized in the pathway) or purifying new enzymes.

This approach is widely used because it offers a quick view into an organism's metabolism. Genome sequences for many organisms are now available, and reconstruction can be partly or fully automated with tools. For example, the tool IMG/M has over 120 000 genomes for bacteria alone, and it displays predicted biochemical reactions in Kyoto Encyclopedia of Genes and Genomes pathways (Chen et al. 2021). Recently, a tool was introduced for reconstructing fermentation pathways specifically (Hackmann and Zhang 2023). With these tools, hundreds or thousands of organisms can be examined at once—a scale unimaginable with traditional biochemistry.

This approach has pitfalls, however. Pathways can have gaps due to a failure at any step of reconstruction. In one study (Hackmann and Zhang 2023), it was found that 61% of butyrate-forming organisms were apparently missing at least one reaction-forming butyrate. Poor gene annotation was likely responsible in the case of one reaction (butyryl CoA:acetate CoA transferase, EC 2.8.3.8); databases are missing annotations for 86% of the enzymes known to catalyze it (Hackmann 2022). Many investigators simply fill these gaps by adding missing reactions back to a reconstructed pathway; this was done in reconstructing pathways of human gut bacteria (Magnusdottir et al. 2017, Heinken et al. 2023). In the end, only experiments can show if reactions are truly missing from an organism, and these experiments can lead to the discovery of more enzymes and pathways.

### Emerging roles for electron bifurcation and confurcation

**Figure 9.**
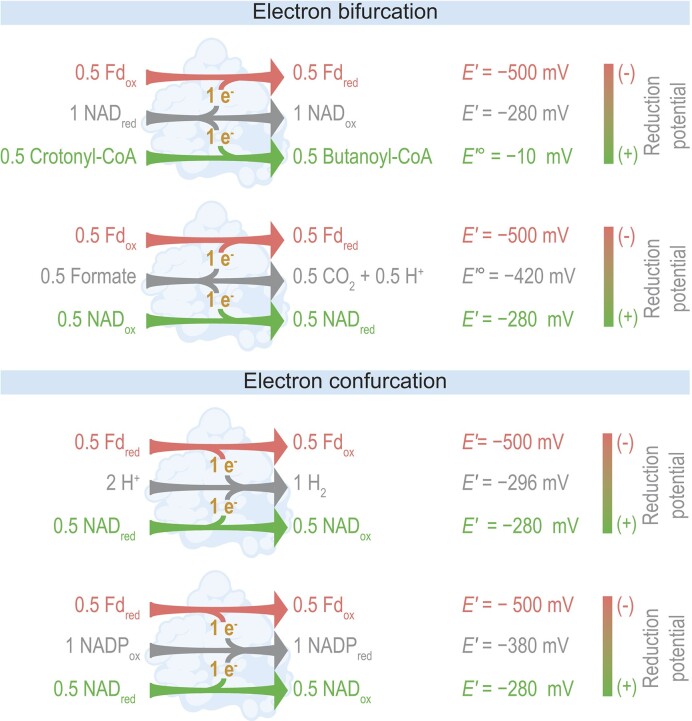
Flavin-based electron bifurcation and electron confurcation are types of energetic coupling important in fermentation. Enzymes shown are butyryl-CoA dehydrogenase (EC 1.3.8.1), formate dehydrogenase (NAD^+^, ferredoxin) (EC 1.17.1.11), hydrogenase (NAD^+^, ferredoxin) (EC 1.12.1.4), and NAD(P)^+^ transhydrogenase (ferredoxin) (Nfn) (EC 1.6.1.4). See Figs. 4, 5, 7 for their role in fermentation. Apparent reduction potentials (*E*′) are from Buckel and Thauer (2013). Values of standard apparent reduction potential (*E*′°) are shown when values of *E*′ are not available. Values are for the half-reaction in the direction of reduction. Reduced ferredoxin refers to two reduced iron–sulfur clusters. CoA = coenzyme A, Fd = ferredoxin, ox = oxidized, and red = reduced.

Some enzymes revealed by recent studies carry out electron bifurcation (Fig. 9), a unique form of energetic coupling (Buckel and Thauer 2018). This coupling involves the transfer of electrons from one donor molecule to two acceptors. One enzyme carrying out this coupling is butyryl-CoA dehydrogenase, which transfers electrons from reduced NAD to oxidized ferredoxin and crotonyl-CoA (Figs. 4, 7, 9) (Jayamani 2008, Li et al. 2008, Demmer et al. 2017). A related process, electron confurcation, involves the transfer of electrons from two donor molecules to one acceptor (Fig. 9). Bifurcating and confurcating enzymes often transfer electrons between ferredoxin and NAD, giving them a key role in balancing these redox cofactors.

While these enzymes balance redox, they have received attention also because of their unique energetics (Buckel and Thauer 2018). During electron bifurcation, an electron is donated to a low-potential acceptor (Fig. 9), a normally unfavorable reaction. This is made possible by donating another electron to a high-potential acceptor (Fig. 9), which is highly favorable. Electron confurcation involves a similar coupling of favorable and unfavorable reactions. Key cofactors in these reactions are flavins (FAD or FMN), which initially accept electrons and then donate them to the final acceptors. As such, these reactions are called flavin-based, distinguishing them from quinone-based reactions found in mitochondria (Buckel and Thauer 2018).

Currently, four flavin-based bifurcating or confurcating enzymes are known to have a role in fermentation (Fig. 9). However, there are more likely to be discovered. First, there are enzymes characterized in non-fermentative organisms that also likely have a role in fermentation. An example is lactate dehydrogenase (NAD^+^, ferredoxin) (EC 1.1.1.436), which transfers electrons from lactate and reduced ferredoxin to oxidized NAD. This enzyme was discovered in a homoacetogen (Weghoff et al. 2015), but genes for it are also present in organisms that ferment lactate (Buckel and Thauer 2018, Detman et al. 2019, Shetty et al. 2020). Second, as one recent study suggests, there could be enzymes not yet characterized in any organism. This study (Schut et al. 2022) searched microbes for genes of the Bfu family of bifurcating enzymes. It found several putative enzymes, and based on sequence homology, they were predicted to use novel electron donors, such as pyruvate (Schut et al. 2022). If proven by further experiments, these enzymes could catalyze reactions previously unknown in fermentation.

### There are additional directions for revealing pathways and enzymes

There are two areas of research to watch in the field of fermentation biochemistry. One is the determination of the 3D structure of fermentation enzymes. In recent years, structures have been determined for several enzymes, including Nfn (Demmer et al. 2015), butyryl-CoA dehydrogenase (Demmer et al. 2017), Mbh (Yu et al. 2018), Rnf (Vitt et al. 2022), hydrogenase (NAD^+^, ferredoxin) (Feng et al. 2022, Furlan et al. 2022, Katsyv et al. 2023), and formate dehydrogenase (hydrogenase) (EC 1.17.98.4) (Steinhilper et al. 2022). These structures were determined through cryo-electron microscopy. Given the increasing application of this technique, more structures can be expected. The structures of these enzymes are key to engineering fermentation to improve catalysis or change reaction substrates.

A second area to watch is reconstruction of fermentation pathways in uncultured microbes. With the availability of genomes of uncultured microbes (Nayfach et al. 2021) and a tool for reconstructing fermentation pathways (Hackmann and Zhang 2023), it should be possible to explore these microbes in greater detail. One roadblock is that many genomes from uncultured microbes have low completeness, leading to gaps in reconstructed pathways. However, improvements in DNA sequencing, genome assembly, and manual curation should lead to more complete genomes, lifting this roadblock (Chen et al. 2020).

## Discoveries in fermentation have real-world applications

Fermentation is not just a fascinating topic to study scientifically, but it has several roles in human society. Agriculture, human health, and industrial production of chemicals are just some areas of society that intersect with fermentation. These areas can be advanced by leveraging fermentative microbes and pathways uncovered by the work above (Fig. S4).

### Engineering microbes can improve the production of commodity chemicals

One application of recent findings is to improve the production of commodity chemicals (Fig. S4). Butanol, succinate, and propionate are among many chemicals produced by fermentation that have industrial uses (Lee et al. 2019, Keasling et al. 2021, Varghese et al. 2022). However, the purity and yield of fermentation products are low (Varghese et al. 2022), and these chemicals are typically produced from petroleum rather than fermentation. One chemical, ethanol, is already produced in large quantities by fermentation (Renewable Fuels Association 2024). However, the current method of production involves fermentation of sugar or starch (Zabed et al. 2017). It would be a vast improvement if it could instead use cellulose, a more abundant resource. Genetic engineering has been long used to improve biological routes for chemical production (Montaño López et al. 2022), and recent findings can help these efforts.

One chemical for which production can be improved is propionate, a food preservative. One barrier to producing propionate by fermentation is that acetate is often formed as an unwanted byproduct. One study tried to engineer a bacterium to produce less acetate by deleting acetate kinase (Suwannakham et al. 2006). While this approach did increase the yield of propionate, the increase (13%) was modest. Later work revealed this bacterium does not use acetate kinase, but rather the two enzymes of the pathway in Fig. 4A (Zhang et al. 2021). These enzymes may be more appropriate targets to increase the yield of the target chemical.

Recent findings are also relevant to the production of the biofuel butanol. The discovery of two new enzymes in its production (Foulquier et al. 2022) (Fig. 4B) identifies additional targets for engineering. While one enzyme (butyryl-CoA dehydrogenase) was previously known in a different role, the second enzyme was previously unknown and represents a completely new target. Overexpressing these two enzymes in *C. acetobutylicum* increased the yield of butanol (Foulquier et al. 2022). This could make butanol production from fermentation more competitive with production from petroleum.

Recent findings could also help realize the goal of producing ethanol from cellulose. *Clostridium thermocellum* can convert cellulose to ethanol, but it forms acetate as an unwanted byproduct. Recent studies (Tian et al. 2016, Lo et al. 2017) suggested that a pathway that forms ethanol is theoretically possible, just as in *T. saccharolyticum* (Fig. 5B). To try to realize this pathway, ferredoxin—NAD^+^ reductase from *T. saccharolyticum* was expressed, and it increased yield of ethanol by 30% (Tian et al. 2016). A second approach, which involved overexpressing Rnf and deleting hydrogenases, increased yield by up to 90% (Lo et al. 2017). This case shows the importance of recently discovered enzymes for balancing redox cofactors, and it underscores they are prime targets for genetic engineering.

Though many studies used genetic engineering, there are other approaches to controlling fermentation that could be informed by recent findings. Electrofermentation is an approach to control fermentation with an electrode that donates or accepts electrons (Moscoviz et al. 2016). It has been used to the increase yield and purity of several chemicals, including propionate (Emde and Schink 1990, Schuppert et al. 1992) and butanol (Kim and Kim 1988, Choi et al. 2014). The mechanism is not fully clear, but it may work by changing the ratio of reduced and oxidized cofactors (Moscoviz et al. 2016). Recently discovered enzymes that balance redox cofactors could thus be relevant. Ferredoxin—NAD^+^ reductase, an enzyme that balances redox cofactors in *C. acetobutylicum* (Fig. 4B), had already been thought to play a role in electrofermentation with this organism (Kim and Kim 1988). Now that this enzyme has been purified and characterized (Foulquier et al. 2022) (Fig. 4B), its exact role can be determined, helping improve the electrofermentation process.

### Manipulating fermentation in the gut could improve human health

Advances in fermentation are also important to improving human health (Fig. S4). Microbes in the gut form several end products (Magnusdottir et al. 2017, Heinken et al. 2023), and many have purported health benefits (Koh et al. 2016, Krautkramer et al. 2021, de Vos et al. 2022). Butyrate, a short-chain fatty acid and product of fiber fermentation, may lower the risk of colorectal cancer. This cancer is relatively rare in people who consume diets high in fiber (Burkitt 1971, Aune et al. 2011), and butyrate appears to promote the growth of normal cells over cancerous ones (Koh et al. 2016). High-fiber diets and butyrate also alleviate metabolic diseases, such as type 2 diabetes (Zhao et al. 2018, Reynolds et al. 2020). Butyrate likely works by activating a cell receptor that raises the production of gut hormones (Tolhurst et al. 2012) and lowers accumulation of fat (Kimura et al. 2013). Acetate and propionate, other products of fiber fermentation, activate this receptor even more strongly (Brown et al. 2003, Le Poul et al. 2003, Nilsson et al. 2003) and maybe even more important. Accordingly, there is a strong push to boost the production of butyrate and other products of fiber fermentation in the gut.

To boost the production of these products, the microbes and enzymes forming them are logical targets. Recent studies identifying these microbes (Fig. 2) and enzymes (Figs. 4, 5, 7) are thus important. Microbes identified to form butyrate could be fed as probiotics, for example. An online tool for identifying microbes by fermentation product could be useful in this regard (Hackmann and Zhang 2023). Genetic engineering is another approach to raise the production of butyrate, and recently discovered enzymes could serve as targets (see Figs. 4,  7).

Despite the attention on products of fiber fermentation, other fermentation products have roles in human health. In a recent study, the end product indole-3-propionate was found to help the host recover from nerve injury (Serger et al. 2022). This end product is formed during amino acid fermentation of *C. sporogenes*, the pathways of which have been recently studied (Fig. 7A). If the yield of this product could be increased, such as with probiotics or genetic engineering, there may be a potential health benefit.

### Improving fermentation is important to many sectors of agriculture

A final application of recent findings is in improving agricultural production (Fig. S4). Cattle and other herbivores ferment their fibrous diet to acetate, propionate, butyrate, and other end products. These end products are absorbed by the host and meet up to 70% of its energy requirements (Bergman 1990). Beyond serving as a source of energy, fermentation products have specific roles in the metabolism of the host. Acetate is a precursor for fatty acids, and infusing it in the gut increases the production of milk fat in cattle (Urrutia and Harvatine 2017). At the same time, hydrogen and formate from fermentation are precursors for methane, a greenhouse gas (Evans et al. 2019). Thus, there is a critical need to manipulate fermentation to maximize the release of energy and animal performance while minimizing the release of greenhouse gases. Microbes, pathways, and enzymes identified in recent studies are all potential targets for achieving this goal.

Another use of fermentation in agriculture is the production of fermented food. This is the oldest use of fermentation, with earliest evidence (malted and fermented seeds) dating to 13 000 years before the present (Liu et al. 2018). In modern times, fermentation is used in the production of roughly 5000 different foods (Tamang 2010). Recent findings could help tailor the production of these foods and select desirable end products. In many cheeses and sausages, the flavor of lactate is desirable but acetate is not (Hutkins 2019). Recent studies could be used to pinpoint organisms that form these products and the pathways they use. They could guide the replacement of heterolactic organisms (ones producing acetate as a byproduct) with homolactic ones (ones producing lactate alone). In foods like sourdough bread, the flavor produced by heterolactic organisms is desired (Hutkins 2019), and fermentation could be tailored in the opposite direction. This type of control is especially important as locally fermented foods are scaled up for global production and starter cultures must be developed.

### Roadblocks must be overcome to improve fermentation

There are roadblocks to overcome before we can improve fermentation for applications outlined in Fig. S4. One roadblock is we lack tools to genetically engineer most microbes. Genetic tools were developed for single, isolated species in culture, but most microbes are uncultured and live in communities with thousands of species (Nayfach et al. 2021). This roadblock has been partly lifted by recent studies employing CRISPR-guided transposons (Vo et al. 2021, Rubin et al. 2022), which can edit bacteria in their native communities. This opens up the possibility that many species, including uncultured ones, can be engineered at once. While a major advance, it still requires organisms to be genetically tractable. In the human gut, the only organisms identified as genetically tractable were strains of *E. coli* (Rubin et al. 2022). More work will need to be done to target other bacteria of the gut and other environments. Given recent progress in making non-model bacteria genetically tractable (Chen et al. 2023, Volke et al. 2023), this limitation may not last long.

Administering probiotics is another approach to improve fermentation, but it also has roadblocks to use. Most probiotics currently available are lactic acid bacteria (O'Toole et al. 2017). They are oxygen-tolerant and easy to manufacture (O'Toole et al. 2017), but a wider range of organisms is needed to tailor fermentation precisely. A recent study developed a method to the increase oxygen tolerance of probiotics (Khan et al. 2023), helping lift barriers to manufacturing. More probiotic formulations can now be tested, which is crucial given current formulations seldom improve health outcomes (Suez et al. 2019, Gilijamse et al. 2020, Khan et al. 2023).

One approach with relatively few roadblocks for controlling fermentation is enzyme inhibitors. The potential of this strategy is illustrated by 3-nitroxypropanol, an inhibitor of an enzyme forming methane (methyl-CoM reductase; EC 2.8.4.1). This inhibitor was found through a virtual screen of compounds against the 3D structure of the target enzyme (Duin et al. 2016). It has been effective in vivo and found to reduce methane emissions of cattle by 30% (Hristov et al. 2015). Similar inhibitors could be developed against fermentation enzymes and reduce unwanted fermentation products. Such inhibitors could even be used to further reduce methane; they could target enzymes that produce hydrogen or formate, precursors for methane. Another approach with few roadblocks is electrofermentation, though its reliance on electrodes limits application in humans or animals.

### Concluding remarks and future perspectives

The study of fermentation has a rich history, but recent studies have highlighted gaps in our knowledge. It is now clear that this metabolism can be found throughout the tree of life. It is also clear that fermentation is complex, forming nearly 300 combinations of products just in the organisms that have been studied. At the biochemical level, enzymes for fermentation continue to be discovered, including old enzymes in new roles. Enzymes for balancing redox cofactors especially have risen to prominence. These enzymes, along with associated pathways and microbes, are key targets for improving fermentation. Agriculture, human health, and industrial production of chemicals are all areas that stand to benefit from recent discoveries.

Recent studies also reveal how much more we have to learn. Fermentation has been best studied in cultured bacteria and archaea, and eukaryotes and uncultured microbes have received far less attention. Engineering microbes for better fermentation is a laudable goal, but it is still challenging to achieve this in communities with thousands of microbes. The most exciting discoveries and breakthroughs in fermentation are still to come.

## Supplementary Material

fuae016_Supplemental_File
