# Supplementary material for: The vast landscape of carbohydrate fermentation in prokaryotes
Source: FEMS Microbiol Rev. 2024 May 31;48(4):fuae016. doi: 10.1093/femsre/fuae016 (PMC11187502; doi:10.1093/femsre/fuae016)
Supplement: fuae016_Supplemental_File [file fuae016_supplemental_file.pdf]

Supplementary Materials for

The Vast Landscape of Carbohydrate Fermentation in Prokaryotes

Timothy J. Hackmann*

*Corresponding author. Department of Animal Science, University of California, Davis, CA 95616, USA. Email: tjhackmann@ucdavis.edu

This PDF file includes

Table S1
Figures S1 to S4

Table S1. Enzyme commission (EC) number and names of enzymes in pathways of fermentation of glucose[1,2]

Module	Reaction	Enzyme	Name
Glycolysis	2_Phospho_D_glycerate <=> 3_Phospho_D_glycerate	5.4.2.11	phosphoglycerate mutase (2,3-diphosphoglycerate-dependent)
Glycolysis	2_Phospho_D_glycerate <=> 3_Phospho_D_glycerate	5.4.2.12	phosphoglycerate mutase (2,3-diphosphoglycerate-independent)
Glycolysis	2_Phospho_D_glycerate <=> Phosphoenolpyruvate + H2O	4.2.1.11	phosphopyruvate hydratase
Glycolysis	ADP + D_Glucose <=> AMP + D_Glucose_6_phosphate	2.7.1.147	ADP-specific glucose/glucosamine kinase
Glycolysis	ATP + 3_Phospho_D_glycerate <=> ADP + 3_Phospho_D_glyceroyl_phosphate	2.7.2.3	phosphoglycerate kinase
Glycolysis	ATP + D_Fructose_6_phosphate <=> ADP + D_Fructose_1_6_bisphosphate	2.7.1.11	6-phosphofructokinase
Glycolysis	ATP + D_Glucose <=> ADP + D_Glucose_6_phosphate	2.7.1.1	hexokinase
Glycolysis	ATP + D_Glucose <=> ADP + D_Glucose_6_phosphate	2.7.1.2	glucokinase
Glycolysis	ATP + Polyphosphate <=> ADP + Polyphosphate	2.7.4.1	ATP-polyphosphate phosphotransferase
Glycolysis	ATP + Pyruvate + Orthophosphate <=> AMP + Phosphoenolpyruvate + Diphosphate	2.7.9.1	pyruvate, phosphate dikinase
Glycolysis	ATP + Pyruvate <=> ADP + Phosphoenolpyruvate	2.7.1.40	pyruvate kinase
Glycolysis	D_Fructose_1_6_bisphosphate <=> Glycerone_phosphate + D_Glyceraldehyde_3_phosphate	4.1.2.13	fructose-bisphosphate aldolase
Glycolysis	D_Glucose_6_phosphate <=> D_Fructose_6_phosphate	5.3.1.9	glucose-6-phosphate isomerase
Glycolysis	D_Glyceraldehyde_3_phosphate + H2O + Oxidized_ferredoxin <=> 3_Phospho_D_glycerate + 2 H + Reduced_ferredoxin	1.2.7.6	glyceraldehyde-3-phosphate dehydrogenase (ferredoxin)
Glycolysis	D_Glyceraldehyde_3_phosphate + Orthophosphate + NAD <=> 3_Phospho_D_glyceroyl_phosphate + NADH + H	1.2.1.12	glyceraldehyde-3-phosphate dehydrogenase (phosphorylating)
Glycolysis	D_Glyceraldehyde_3_phosphate + Orthophosphate + NAD <=> 3_Phospho_D_glyceroyl_phosphate + NADH + H	1.2.1.59	glyceraldehyde-3-phosphate dehydrogenase (NAD(P)+) (phosphorylating)
Glycolysis	D_Glyceraldehyde_3_phosphate + Orthophosphate + NADP <=> 3_Phospho_D_glyceroyl_phosphate + NADPH + H	1.2.1.59	glyceraldehyde-3-phosphate dehydrogenase (NAD(P)+) (phosphorylating)
Glycolysis	D_Glyceraldehyde_3_phosphate <=> Glycerone_phosphate	5.3.1.1	triose-phosphate isomerase
Glycolysis	Diphosphate + D_Fructose_6_phosphate <=> Orthophosphate + D_Fructose_1_6_bisphosphate	2.7.1.90	diphosphate---fructose-6-phosphate 1-phosphotransferase
Glycolysis	Phosphoenolpyruvate + Protein_histidine <=> Pyruvate + Protein_N(pi)_phospho_L_histidine	2.7.3.9	phosphoenolpyruvate---protein phosphotransferase
Glycolysis	Polyphosphate + D_Glucose <=> Polyphosphate + D_Glucose_6_phosphate	2.7.1.63	polyphosphate---glucose phosphotransferase
Glycolysis	Protein_N(pi)_phospho_L_histidine + D_Glucose <=> Protein_histidine + D_Glucose_6_phosphate	2.7.1.199	protein-Npi-phosphohistidine---D-glucose phosphotransferase
Pentose phosphate pathway	2_Dehydro_3_deoxy_6_phospho_D_gluconate <=> D_Glyceraldehyde_3_phosphate + Pyruvate	4.1.2.14	2-dehydro-3-deoxy-phosphogluconate aldolase
Pentose phosphate pathway	2_Dehydro_3_deoxy_6_phospho_D_gluconate <=> D_Glyceraldehyde_3_phosphate + Pyruvate	4.1.2.55	2-dehydro-3-deoxy-phosphogluconate/2-dehydro-3-deoxy-6-phosphogalactonate aldolase
Pentose phosphate pathway	2_Dehydro_3_deoxy_D_gluconate <=> D_Glyceraldehyde + Pyruvate	4.1.2.51	2-dehydro-3-deoxy-D-gluconate aldolase
Pentose phosphate pathway	2_Dehydro_3_deoxy_D_gluconate <=> D_Glyceraldehyde + Pyruvate	4.1.2.55	2-dehydro-3-deoxy-phosphogluconate/2-dehydro-3-deoxy-6-phosphogalactonate aldolase
Pentose phosphate pathway	6_Phospho_D_gluconate + NAD <=> 6_Phospho_2_dehydro_D_gluconate + NADH + H	1.1.1.43	phosphogluconate 2-dehydrogenase
Pentose phosphate pathway	6_Phospho_D_gluconate + NAD <=> D_Ribulose_5_phosphate + CO2 + NADH + H	1.1.1.343	phosphogluconate dehydrogenase (NAD+-dependent, decarboxylating)
Pentose phosphate pathway	6_Phospho_D_gluconate + NAD <=> D_Ribulose_5_phosphate + CO2 + NADH + H	1.1.1.351	phosphogluconate dehydrogenase [NAD(P)+-dependent, decarboxylating]
Pentose phosphate pathway	6_Phospho_D_gluconate + NADP <=> D_Ribulose_5_phosphate + CO2 + NADPH + H	1.1.1.351	phosphogluconate dehydrogenase [NAD(P)+-dependent, decarboxylating]
Pentose phosphate pathway	6_Phospho_D_gluconate + NADP <=> D_Ribulose_5_phosphate + CO2 + NADPH + H	1.1.1.44	phosphogluconate dehydrogenase (NADP+-dependent, decarboxylating)
Pentose phosphate pathway	6_Phospho_D_gluconate <=> 2_Dehydro_3_deoxy_6_phospho_D_gluconate + H2O	4.2.1.12	phosphogluconate dehydratase
Pentose phosphate pathway	ATP + 2_Dehydro_3_deoxy_D_gluconate <=> ADP + 2_Dehydro_3_deoxy_6_phospho_D_gluconate	2.7.1.45	2-dehydro-3-deoxygluconokinase
Pentose phosphate pathway	ATP + 2_Keto_D_gluconic_acid <=> ADP + 6_Phospho_2_dehydro_D_gluconate	2.7.1.13	dehydrogluconokinase
Pentose phosphate pathway	D_Fructose_6_phosphate + D_Glyceraldehyde_3_phosphate <=> D_Erythrose_4_phosphate + D_Xylulose_5_phosphate	2.2.1.1	transketolase
Pentose phosphate pathway	D_Fructose_6_phosphate + Orthophosphate <=> Acetyl_phosphate + D_Erythrose_4_phosphate + H2O	4.1.2.22	fructose-6-phosphate phosphoketolase
Pentose phosphate pathway	D_Gluconic_acid + NADP <=> 2_Keto_D_gluconic_acid + NADPH + H	1.1.1.215	gluconate 2-dehydrogenase
Pentose phosphate pathway	D_Gluconic_acid <=> 2_Dehydro_3_deoxy_D_gluconate + H2O	4.2.1.140	gluconate/galactonate dehydratase
Pentose phosphate pathway	D_Glucono_1_5_lactone + H2O <=> D_Gluconic_acid	3.1.1.17	gluconolactonase
Pentose phosphate pathway	D_Glucono_1_5_lactone_6_phosphate + H2O <=> 6_Phospho_D_gluconate	3.1.1.31	6-phosphogluconolactonase
Pentose phosphate pathway	D_Glucose + NAD <=> D_Glucono_1_5_lactone + NADH + H	1.1.1.359	aldose 1-dehydrogenase [NAD(P)+]
Pentose phosphate pathway	D_Glucose + NAD <=> D_Glucono_1_5_lactone + NADH + H	1.1.1.47	glucose 1-dehydrogenase [NAD(P)+]
Pentose phosphate pathway	D_Glucose + NADP <=> D_Glucono_1_5_lactone + NADPH + H	1.1.1.359	aldose 1-dehydrogenase [NAD(P)+]
Pentose phosphate pathway	D_Glucose + NADP <=> D_Glucono_1_5_lactone + NADPH + H	1.1.1.47	glucose 1-dehydrogenase [NAD(P)+]
Pentose phosphate pathway	D_Glucose_6_phosphate + NAD <=> D_Glucono_1_5_lactone_6_phosphate + NADH + H	1.1.1.363	glucose-6-phosphate dehydrogenase [NAD(P)+]
Pentose phosphate pathway	D_Glucose_6_phosphate + NAD <=> D_Glucono_1_5_lactone_6_phosphate + NADH + H	1.1.1.388	glucose-6-phosphate dehydrogenase (NAD+)
Pentose phosphate pathway	D_Glucose_6_phosphate + NADP <=> D_Glucono_1_5_lactone_6_phosphate + NADPH + H	1.1.1.363	glucose-6-phosphate dehydrogenase [NAD(P)+]
Pentose phosphate pathway	D_Glucose_6_phosphate + NADP <=> D_Glucono_1_5_lactone_6_phosphate + NADPH + H	1.1.1.49	glucose-6-phosphate dehydrogenase (NADP+)
Pentose phosphate pathway	D_Glucose_6_phosphate <=> D_Fructose_6_phosphate	5.3.1.9	glucose-6-phosphate isomerase
Pentose phosphate pathway	D_Glyceraldehyde + H2O + Oxidized_ferredoxin <=> D_Glycerate + H + Reduced_ferredoxin	1.2.7.5	aldehyde ferredoxin oxidoreductase
Pentose phosphate pathway	D_Glycerate + ATP <=> 2_Phospho_D_glycerate + ADP	2.7.1.165	glycerate 2-kinase
Pentose phosphate pathway	D_Ribose_5_phosphate <=> D_Ribulose_5_phosphate	5.3.1.6	ribose-5-phosphate isomerase
Pentose phosphate pathway	D_Ribulose_5_phosphate <=> D_Xylulose_5_phosphate	5.1.3.1	ribulose-phosphate 3-epimerase
Pentose phosphate pathway	D_Xylulose_5_phosphate + Orthophosphate <=> Acetyl_phosphate + D_Glyceraldehyde_3_phosphate + H2O	4.1.2.9	phosphoketolase
Pentose phosphate pathway	Sedoheptulose_7_phosphate + D_Glyceraldehyde_3_phosphate <=> D_Erythrose_4_phosphate + D_Fructose_6_phosphate	2.2.1.2	transaldolase
Pentose phosphate pathway	Sedoheptulose_7_phosphate + D_Glyceraldehyde_3_phosphate <=> D_Ribose_5_phosphate + D_Xylulose_5_phosphate	2.2.1.1	transketolase
Methylglyoxal shunt	(R)_Lactaldehyde + NAD + H2O <=> (R)_Lactate + NADH + H	1.2.1.23	2-oxoaldehyde dehydrogenase (NAD+)
Methylglyoxal shunt	(R)_Lactaldehyde + NAD <=> Methylglyoxal + NADH + H	1.1.1.78	methylglyoxal reductase (NADH)
Methylglyoxal shunt	(R)_Lactate + NAD <=> Pyruvate + NADH + H	1.1.1.28	D-lactate dehydrogenase
Methylglyoxal shunt	(R)_Lactate <=> Methylglyoxal + H2O	4.2.1.130	D-lactate dehydratase
Methylglyoxal shunt	(R)_S_Lactoylglutathione + H2O <=> Glutathione + (R)_Lactate	3.1.2.6	hydroxyacylglutathione hydrolase
Methylglyoxal shunt	(R)_S_Lactoylglutathione <=> Glutathione + Methylglyoxal	4.4.1.5	lactoylglutathione lyase
Methylglyoxal shunt	(S)_Lactaldehyde + NAD + H2O <=> (S)_Lactate + NADH + H	1.2.1.22	lactaldehyde dehydrogenase
Methylglyoxal shunt	(S)_Lactaldehyde + NAD <=> Methylglyoxal + NADH + H	1.1.1.6	glycerol dehydrogenase
Methylglyoxal shunt	(S)_Lactate + NAD <=> Pyruvate + NADH + H	1.1.1.27	L-lactate dehydrogenase
Methylglyoxal shunt	D_Glyceraldehyde_3_phosphate <=> Glycerone_phosphate	5.3.1.1	triose-phosphate isomerase
Methylglyoxal shunt	Glycerone_phosphate <=> Methylglyoxal + Orthophosphate	4.2.3.3	methylglyoxal synthase
Methylglyoxal shunt	Methylglyoxal + NAD + H2O <=> Pyruvate + NADH + H	1.2.1.22	lactaldehyde dehydrogenase
Methylglyoxal shunt	Methylglyoxal + NAD + H2O <=> Pyruvate + NADH + H	1.2.1.23	2-oxoaldehyde dehydrogenase (NAD+)
Malate shunt	(S)_Malate + NAD <=> Oxaloacetate + NADH + H	1.1.1.37	malate dehydrogenase
Malate shunt	(S)_Malate + NAD <=> Pyruvate + CO2 + NADH + H	1.1.1.38	malate dehydrogenase (oxaloacetate-decarboxylating)
Malate shunt	(S)_Malate + NAD <=> Pyruvate + CO2 + NADH + H	1.1.1.39	malate dehydrogenase (decarboxylating)
Malate shunt	(S)_Malate + NADP <=> Oxaloacetate + NADPH + H	1.1.1.82	malate dehydrogenase (NADP+)
Malate shunt	(S)_Malate + NADP <=> Pyruvate + CO2 + NADPH + H	1.1.1.40	malate dehydrogenase (oxaloacetate-decarboxylating) (NADP+)
Malate shunt	(S)_Methylmalonyl_CoA + Pyruvate <=> Propanoyl_CoA + Oxaloacetate	2.1.3.1	methylmalonyl-CoA carboxytransferase
Malate shunt	ATP + Oxaloacetate <=> ADP + Phosphoenolpyruvate + CO2	4.1.1.49	phosphoenolpyruvate carboxykinase (ATP)
Malate shunt	ATP + Pyruvate + HCO3 <=> ADP + Orthophosphate + Oxaloacetate	6.4.1.1	pyruvate carboxylase
Malate shunt	Diphosphate + Oxaloacetate <=> Orthophosphate + Phosphoenolpyruvate + CO2	4.1.1.38	phosphoenolpyruvate carboxykinase (diphosphate)
Malate shunt	GTP + Oxaloacetate <=> GDP + Phosphoenolpyruvate + CO2	4.1.1.32	phosphoenolpyruvate carboxykinase (GTP)
Malate shunt	Orthophosphate + Oxaloacetate <=> H2O + Phosphoenolpyruvate + CO2	4.1.1.31	phosphoenolpyruvate carboxylase
Malate shunt	Oxaloacetate + 2 Sodium_cation <=> Pyruvate + CO2 + 2 Sodium_cation	7.2.4.2	oxaloacetate decarboxylase (Na+ extruding)
Malate shunt	Oxaloacetate <=> Pyruvate + CO2	1.1.1.38	malate dehydrogenase (oxaloacetate-decarboxylating)
Malate shunt	Oxaloacetate <=> Pyruvate + CO2	1.1.1.40	malate dehydrogenase (oxaloacetate-decarboxylating) (NADP+)
Malate shunt	Oxaloacetate <=> Pyruvate + CO2	4.1.1.112	oxaloacetate decarboxylase
Formate (or ferredoxin) formation	Reduced_ferredoxin + Acetyl_CoA + CO2 + 2 H <=> Oxidized_ferredoxin + Pyruvate + CoA	1.2.7.1	pyruvate synthase
Formate (or ferredoxin) formation	Reduced_ferredoxin + Acetyl_CoA + CO2 + 2 H <=> Oxidized_ferredoxin + Pyruvate + CoA	1.2.7.11	2-oxoacid oxidoreductase (ferredoxin)
Formate (or ferredoxin) formation	Acetyl_CoA + Enzyme_N6_(dihydrolipoyl)lysine <=> CoA + [Dihydrolipoyllysine_residue_acetyltransferase]_S_acetyldihydrolipoyllysine	2.3.1.12	dihydrolipoyllysine-residue acetyltransferase
Formate (or ferredoxin) formation	Acetyl_CoA + Formate <=> CoA + Pyruvate	2.3.1.54	formate C-acetyltransferase
Formate (or ferredoxin) formation	Enzyme_N6_(dihydrolipoyl)lysine + NAD <=> Enzyme_N6_(lipoyl)lysine + NADH + H	1.8.1.4	dihydrolipoyl dehydrogenase
Formate (or ferredoxin) formation	Pyruvate + Enzyme_N6_(lipoyl)lysine <=> [Dihydrolipoyllysine_residue_acetyltransferase]_S_acetyldihydrolipoyllysine + CO2	1.2.4.1	pyruvate dehydrogenase (acetyl-transferring)
Lactate formation	(R)_Lactate + NAD <=> Pyruvate + NADH + H	1.1.1.28	D-lactate dehydrogenase
Lactate formation	(S)_Lactate + NAD <=> Pyruvate + NADH + H	1.1.1.27	L-lactate dehydrogenase
Lactate formation	(S)_Lactate <=> (R)_Lactate	5.1.2.1	lactate racemase
			**CONTINUED**

Table S1: CONTINUED

Ethanol formation	Acetaldehyde + CoA + NAD <=> Acetyl_CoA + NADH + H	1.2.1.10	acetaldehyde dehydrogenase (acetylating)
Ethanol formation	Acetaldehyde + Thiamin_diphosphate <=> 2_(alpha_Hydroxyethyl)thiamine_diphosphate	4.1.1.1	pyruvate decarboxylase
Ethanol formation	Ethanol + NAD <=> Acetaldehyde + NADH + H	1.1.1.1	alcohol dehydrogenase
Ethanol formation	Pyruvate + Thiamin_diphosphate <=> 2_(alpha_Hydroxyethyl)thiamine_diphosphate + CO2	4.1.1.1	pyruvate decarboxylase
Acetate formation	ATP + Acetate + CoA <=> ADP + Acetyl_CoA + Orthophosphate	6.2.1.13	acetate---CoA ligase (ADP-forming)
Acetate formation	ATP + Acetate + CoA <=> AMP + Diphosphate + Acetyl_CoA	6.2.1.1	acetate---CoA ligase
Acetate formation	ATP + Acetate <=> ADP + Acetyl_phosphate	2.7.2.1	acetate kinase
Acetate formation	ATP + Butanoic_acid <=> ADP + Butanoylphosphate	2.7.2.7	butyrate kinase
Acetate formation	ATP + Succinate + CoA <=> ADP + Orthophosphate + Succinyl_CoA	6.2.1.5	succinate---CoA ligase (ADP-forming)
Acetate formation	Acetyl_CoA + Orthophosphate <=> CoA + Acetyl_phosphate	2.3.1.8	phosphate acetyltransferase
Acetate formation	Butanoyl_CoA + Acetate <=> Butanoic_acid + Acetyl_CoA	2.8.3.8	acetate CoA-transferase
Acetate formation	Butanoyl_CoA + Orthophosphate <=> CoA + Butanoylphosphate	2.3.1.19	phosphate butyryltransferase
Acetate formation	GTP + Succinate + CoA <=> GDP + Orthophosphate + Succinyl_CoA	6.2.1.4	succinate---CoA ligase (GDP-forming)
Acetate formation	Pyruvate + Ubiquinone + H2O <=> Acetate + Ubiquinol + CO2	1.2.5.1	pyruvate dehydrogenase (quinone)
Acetate formation	Succinyl_CoA + Acetate <=> Acetyl_CoA + Succinate	2.8.3.18	succinyl-CoA:acetate CoA-transferase
Butyrate formation	(S)_3_Hydroxybutanoyl_CoA + NADP <=> Acetoacetyl_CoA + NADPH + H	1.1.1.157	3-hydroxybutyryl-CoA dehydrogenase
Butyrate formation	(S)_3_Hydroxybutanoyl_CoA <=> Crotonoyl_CoA + H2O	4.2.1.17	enoyl-CoA hydratase
Butyrate formation	2 Acetyl_CoA <=> CoA + Acetoacetyl_CoA	2.3.1.9	acetyl-CoA C-acetyltransferase
Butyrate formation	ATP + Acetate <=> ADP + Acetyl_phosphate	2.7.2.1	acetate kinase
Butyrate formation	ATP + Butanoic_acid <=> ADP + Butanoylphosphate	2.7.2.7	butyrate kinase
Butyrate formation	Acetyl_CoA + Orthophosphate <=> CoA + Acetyl_phosphate	2.3.1.8	phosphate acetyltransferase
Butyrate formation	Butanoyl_CoA + Acetate <=> Butanoic_acid + Acetyl_CoA	2.8.3.8	acetate CoA-transferase
Butyrate formation	Butanoyl_CoA + FAD <=> FADH2 + Crotonoyl_CoA	1.3.8.1	short-chain acyl-CoA dehydrogenase
Butyrate formation	Butanoyl_CoA + Orthophosphate <=> CoA + Butanoylphosphate	2.3.1.19	phosphate butyryltransferase
Propionate/succinate formation	(R)_Methylmalonyl_CoA <=> (S)_Methylmalonyl_CoA	5.1.99.1	methylmalonyl-CoA epimerase
Propionate/succinate formation	(R)_Methylmalonyl_CoA <=> Succinyl_CoA	5.4.99.2	methylmalonyl-CoA mutase
Propionate/succinate formation	(S)_Malate <=> Fumarate + H2O	4.2.1.2	fumarate hydratase
Propionate/succinate formation	(S)_Methylmalonyl_CoA + Na[side_1] + H[side_2] <=> Propanoyl_CoA + CO2 + Na[side_2]	7.2.4.3	(S)-methylmalonyl-CoA decarboxylase (sodium-transporting)
Propionate/succinate formation	(S)_Methylmalonyl_CoA + Pyruvate <=> Propanoyl_CoA + Oxaloacetate	2.1.3.1	methylmalonyl-CoA carboxytransferase
Propionate/succinate formation	(S)_Methylmalonyl_CoA <=> Propanoyl_CoA + CO2	4.1.1.-	methylmalonyl-CoA decarboxylase
Propionate/succinate formation	ATP + Propanoate + CoA <=> ADP + Orthophosphate + Propanoyl_CoA	6.2.1.13	acetate---CoA ligase (ADP-forming)
Propionate/succinate formation	ATP + Propanoate <=> ADP + Propanoyl_phosphate	2.7.2.1	acetate kinase
Propionate/succinate formation	ATP + Succinate + CoA <=> ADP + Orthophosphate + Succinyl_CoA	6.2.1.5	succinate---CoA ligase (ADP-forming)
Propionate/succinate formation	GTP + Succinate + CoA <=> GDP + Orthophosphate + Succinyl_CoA	6.2.1.4	succinate---CoA ligase (GDP-forming)
Propionate/succinate formation	Lactoyl_CoA + Propanoate <=> (S)_Lactate + Propanoyl_CoA	2.8.3.1	propionate CoA-transferase
Propionate/succinate formation	Lactoyl_CoA <=> Propenoyl_CoA + H2O	4.2.1.54	lactoyl-CoA dehydratase
Propionate/succinate formation	Propanoyl_CoA + NAD <=> Propenoyl_CoA + NADH + H	1.3.1.95	acrylyl-CoA reductase (NADH)
Propionate/succinate formation	Propanoyl_CoA + Orthophosphate <=> Propanoyl_phosphate + CoA	2.3.1.8	phosphate acetyltransferase
Propionate/succinate formation	Propanoyl_CoA + Succinate <=> Succinyl_CoA + Propanoate	2.8.3.27	propanoyl-CoA:succinate CoA transferase
Propionate/succinate formation	Quinone + Succinate <=> Hydroquinone + Fumarate	1.3.5.1	succinate dehydrogenase
Hydrogen formation	2 Hydrogen + NAD + Oxidized_ferredoxin <=> 5 H + NADH + Reduced_ferredoxin	1.12.1.4	hydrogenase (NAD+, ferredoxin)
Hydrogen formation	Reduced_ferredoxin + 2 H + 2 H[side_1] <=> Hydrogen + Oxidized_ferredoxin + 2 H[side_2]	7.2.1.-A	Ech hydrogenase
Hydrogen formation	Reduced_ferredoxin + 2 H + Na[side_1] <=> Hydrogen + Oxidized_ferredoxin + Na[side_2]	7.2.1.-B	Mbh hydrogenase
Hydrogen formation	Reduced_ferredoxin + 2 H <=> Hydrogen + Oxidized_ferredoxin	1.12.7.2	ferredoxin hydrogenase
Hydrogen formation	CO2 + Hydrogen <=> Formate	1.17.98.4	formate dehydrogenase (hydrogenase)
Hydrogen formation	Hydrogen + NAD <=> NADH + H	1.12.1.2	hydrogen dehydrogenase
Hydrogen formation	Hydrogen + NAD <=> NADH + H	1.12.1.5	hydrogen dehydrogenase [NAD(P)+]
Hydrogen formation	Hydrogen + NADP <=> NADPH + H	1.12.1.3	hydrogen dehydrogenase (NADP+)
Hydrogen formation	Hydrogen + NADP <=> NADPH + H	1.12.1.5	hydrogen dehydrogenase [NAD(P)+]
Electron transfer	Reduced_ferredoxin + NAD + H + Na[side_1] <=> Oxidized_ferredoxin + NADH + Na[side_2]	7.2.1.2	ferredoxin---NAD+ oxidoreductase (Na+-transporting)
Electron transfer	Reduced_ferredoxin + NAD + H <=> Oxidized_ferredoxin + NADH	1.18.1.3	ferredoxin---NAD+ reductase
Electron transfer	Reduced_ferredoxin + NADP + H <=> Oxidized_ferredoxin + NADPH	1.18.1.2	ferredoxin---NADP+ reductase
Electron transfer	Formate + Quinone <=> CO2 + Hydroquinone	1.17.5.3	formate dehydrogenase-N
Electron transfer	Menaquinone + Hydrogen <=> Menaquinol	1.12.5.1	hydrogen:quinone oxidoreductase
Electron transfer	Menaquinone + Hydrogen <=> Menaquinol	1.12.99.6	hydrogenase (acceptor)
Electron transfer	NADH + H + 2 NADP + Reduced_ferredoxin <=> NAD + 2 NADPH + Oxidized_ferredoxin	1.6.1.4	NAD(P)+ transhydrogenase (ferredoxin)
Electron transfer	NADH + H + Ubiquinone + Na[side_1] <=> NAD + Ubiquinol + Na[side_2]	7.2.1.1	NADH:ubiquinone reductase (Na+-transporting)
Electron transfer	Ubiquinone + NADH + 6 H <=> Ubiquinol + NAD + 5 H	1.6.5.9	NADH:quinone reductase (non-electrogenic)
Electron transfer	Ubiquinone + NADH + 6 H <=> Ubiquinol + NAD + 5 H	7.1.1.2	NADH:ubiquinone reductase (H+-translocating)
ATP formation	ATP + H2O + 4 H[side_1] <=> ADP + Orthophosphate + 4 H[side_2]	7.1.2.2	H+-transporting two-sector ATPase
ATP formation	ATP + H2O + Na[side_1] <=> ADP + Orthophosphate + Na[side_2]	7.2.2.1	Na+-transporting two-sector ATPase
ATP formation	Diphosphate + H2O + Na[side_1] <=> 2 Orthophosphate + Na[side_2]	7.2.3.1	Na+-exporting diphosphatase

[1]Reactions and names are mostly Kyoto Encyclopedia of Genes and Genomes (KEGG) database and from Hackmann and Zhang (2023)

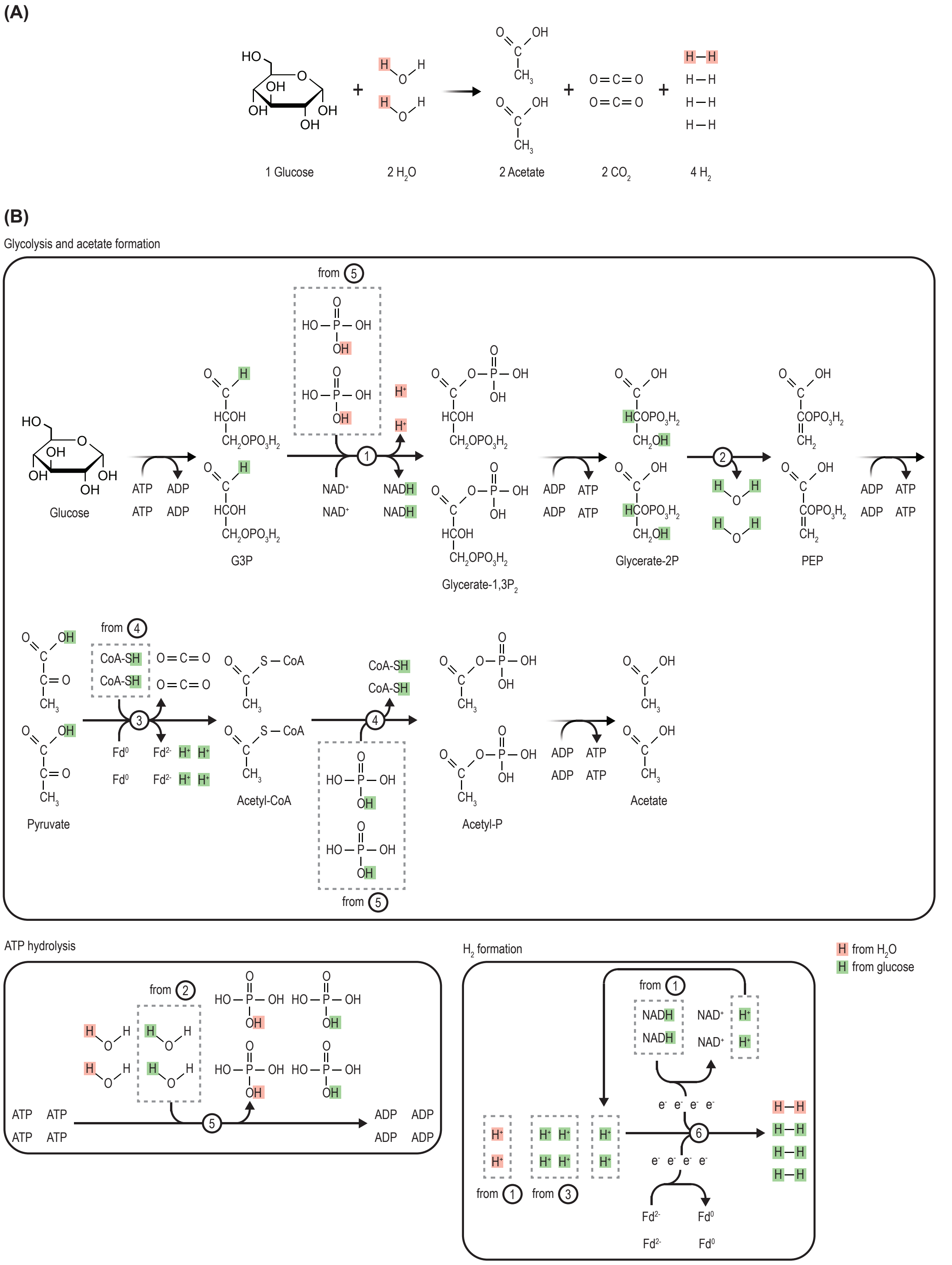

Figure S1. Protons from water can be an electron acceptor during fermentation. (A) Overall reaction of acetate fermentation, showing that H atoms in H_2 originate partly from water. The H atoms from water may be on the same H_2 molecule (as shown) or two separate molecules. (B) Reactions in detail, showing how protons from water are an electron acceptor. Reaction 3 is drawn from information in Ragsdale (2003). Reaction 4 is drawn from information in Lawrence et al. (2006). Reaction 6 is drawn from information in Feng et al. (2022), Furlan et al. (2022), and Katsyv et al. (2023). All other reactions are drawn from information in Nelson and Cox (2021). Electrons are shown explicitly only in reaction 6. H_3PO_4 with H atoms from water can be used at reaction 1 (as shown) or reaction 4. Enzymes: 1, glyceraldehyde-3-phosphate dehydrogenase (phosphorylating) (EC 1.2.1.12); 2, enolase (phosphopyruvate hydratase) (EC 4.2.1.11); 3, pyruvate:ferredoxin oxidoreductase (pyruvate synthase) (EC 1.2.7.1); 4, phosphate acetyltransferase (EC 2.3.1.8); 5, any reaction that hydrolyzes ATP; and 6, hydrogenase (NAD^+, ferredoxin) (EC 1.12.1.4). Abbreviations: -1,3-P2 = 1,3-bisphosphate, -2P = 2-phosphate, -3P = 3-phosphate, CoA = coenzyme A, Fd = ferredoxin, G3P = glyceraldehyde-3P, -P = phosphate, and PEP = phosphoenolpyruvate.

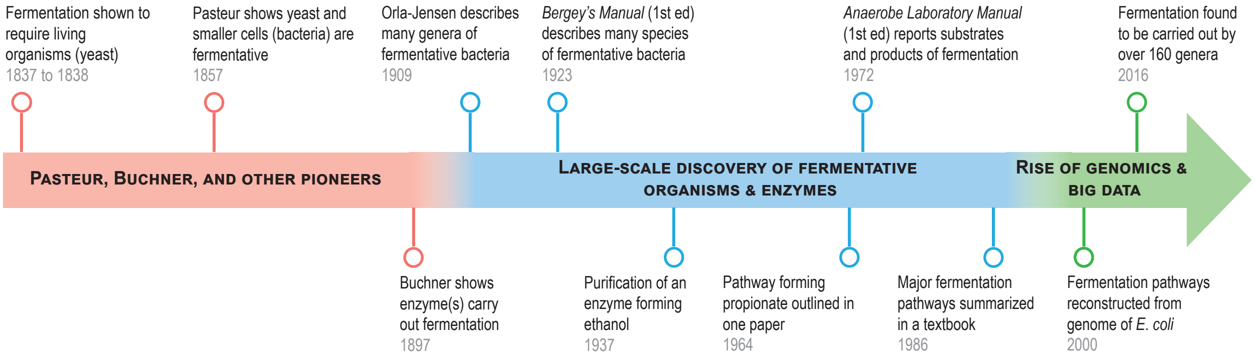

Figure S2. Fermentation research has a long history. For events in 1837 to 1838, see Kützing (1837), Schwann (1837), and Cagniard-Latour (1838); for 1857, see Pastuer (1857a, 1857b); for 1897, see Buchner (1897); for 1909, see Orla-Jensen (1909); for 1923 see Bergey et al. (1923); for 1937, see Negelein and Wulff (1937); for 1964, see Allen et al. (1964); for 1972, see Holdeman and Moore (1972); for 1986, see Gottschalk (1986); for 2000, see Edwards and Palsson (2000); and for 2016, see Louca, Parfrey and Doebeli (2016).

Figure S3. The biochemical pathways of fermentation of glucose in poster format. Drawn from Hackmann *et al.* (2017), Hackmann and Zhang (2023), and references in text. Names of enzymes are in Table S1. See next page for figure.

The biochemical pathways of fermentation of glucose

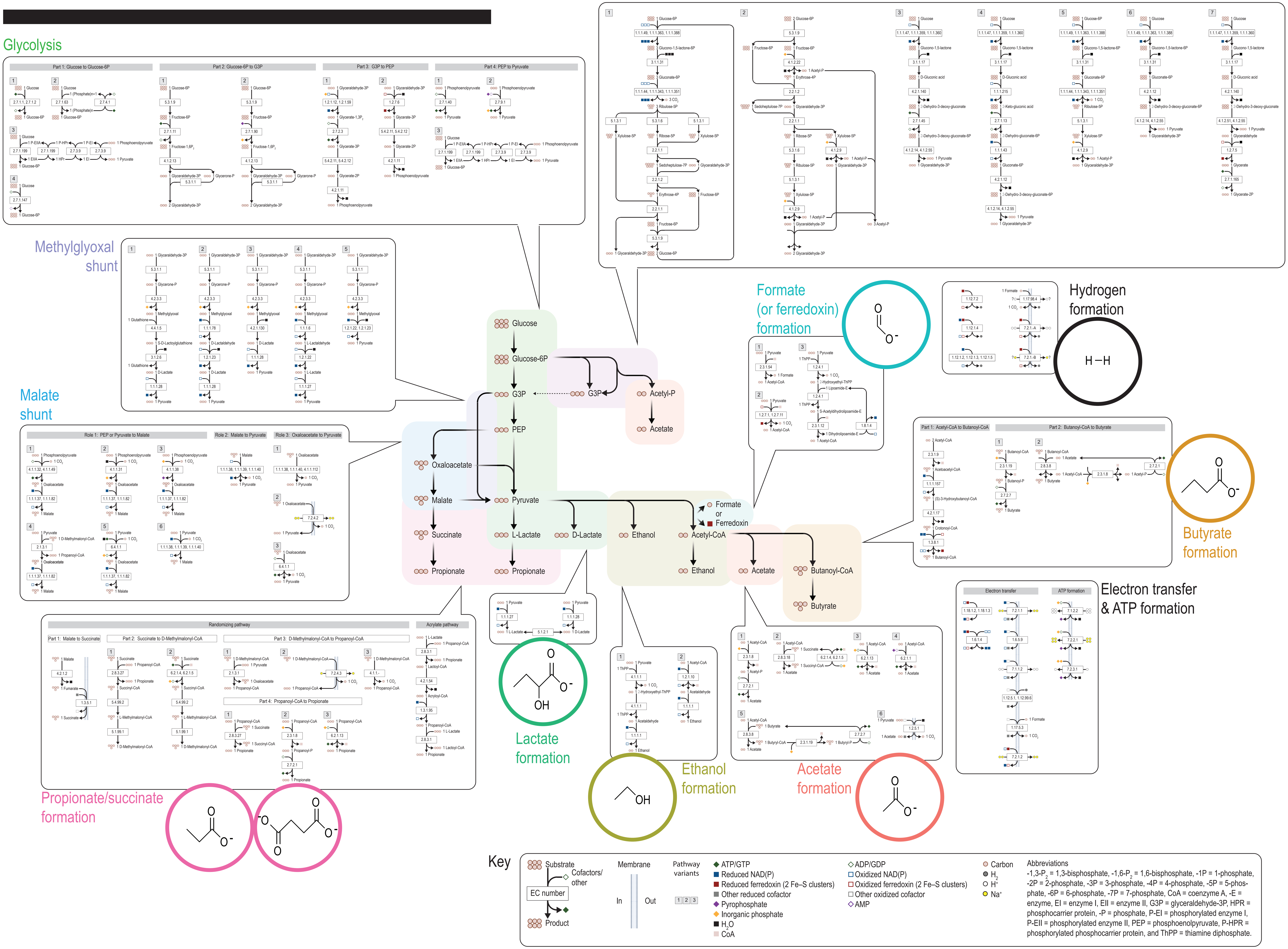

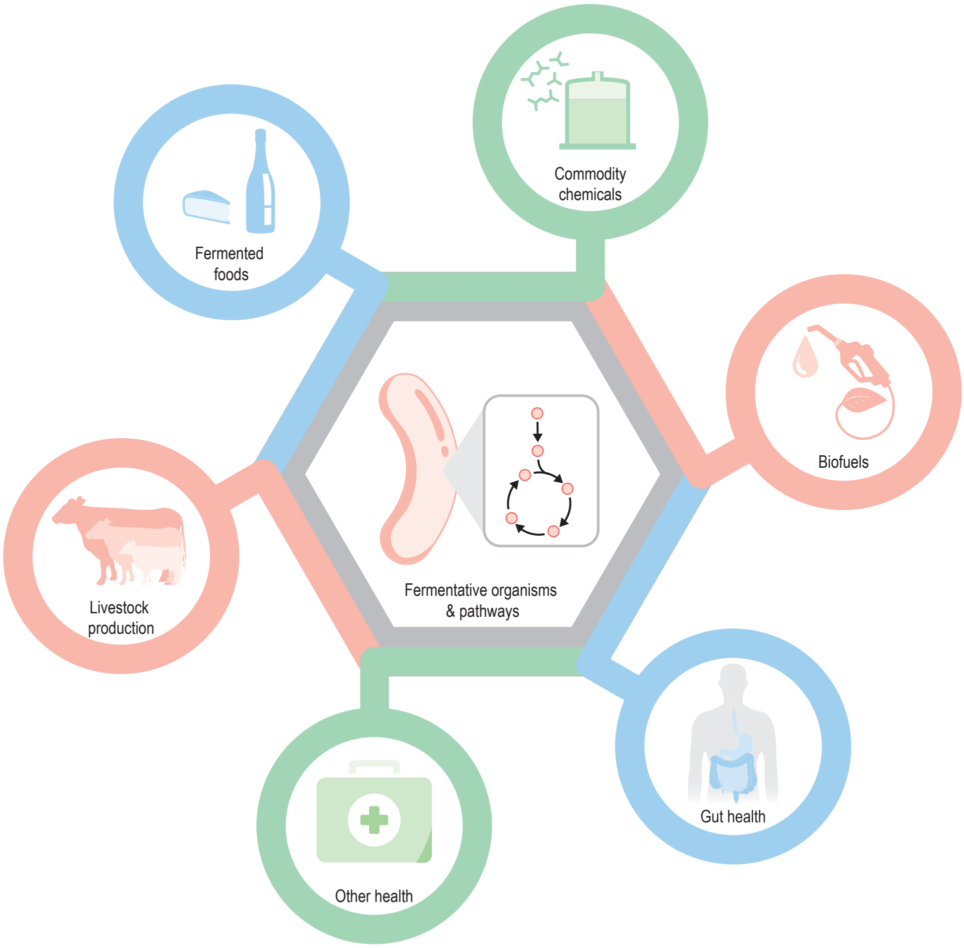

Figure S4. Advances in fermentation research have several practical applications.

References

Allen SH, Kellermeyer RW, Stjernholm RL *et al.* Purification and properties of enzymes involved in the propionic acid fermentation. *J Bacteriol* 1964;**87**:171–87.

Bergey DH, Harrison FC, Breed RS *et al. Bergey's Manual of Determinative Bacteriology*. 1st ed. Williams & Wilkins Company, 1923.

Buchner E. Alkoholische gärung ohne hefezellen. *Ber Dtsch Chem Ges* 1897;**30**:1110–3.

Cagniard-Latour C. Mémoire sur la fermentation vineuse. *Ann Chim Phys* 1838;**68**:206–22.

Edwards JS, Palsson BO. The *Escherichia coli* MG1655 in silico metabolic genotype: its definition, characteristics, and capabilities. *Proc Natl Acad Sci U S A* 2000;**97**:5528–33.

Feng X, Schut GJ, Haja DK *et al.* Structure and electron transfer pathways of an electron-bifurcating NiFe-hydrogenase. *Sci Adv* 2022;**8**:eabm7546.

Furlan C, Chongdar N, Gupta P *et al.* Structural insight on the mechanism of an electron-bifurcating [FeFe] hydrogenase. *eLife* 2022;**11**:e79361.

Gottschalk G. *Bacterial Metabolism*. New York, NY, USA: Springer Verlag, 1986.

Hackmann TJ, Ngugi DK, Firkins JL *et al.* Genomes of rumen bacteria encode atypical pathways for fermenting hexoses to short-chain fatty acids. *Env Microbiol* 2017;**19**:4670–83.

Hackmann TJ, Zhang B. The phenotype and genotype of fermentative prokaryotes. *Sci Adv* 2023;**9**:eadg8687.

Holdeman LV, Moore WEC. *Anaerobe Laboratory Manual*. 1st ed. Blacksburg, Virginia: Virginia Polytechnic Institute and State University, 1972.

Katsyv A, Kumar A, Saura P *et al.* Molecular basis of the electron bifurcation mechanism in the [FeFe]-hydrogenase complex HydABC. *J Am Chem Soc* 2023;**145**:5696–709.

Kützing F. Microscopische Untersuchungen über die Hefe und Essigmutter, nebst mehreren andern dazu gehörigen vegetabilischen Gebilden. *J Für Prakt Chem* 1837;**11**:385–409.

Lawrence SH, Luther KB, Schindelin H *et al.* Structural and functional studies suggest a catalytic mechanism for the phosphotransacetylase from *Methanosarcina thermophila*. *J Bacteriol* 2006;**188**:1143–54.

Louca S, Parfrey LW, Doebeli M. Decoupling function and taxonomy in the global ocean microbiome. *Science* 2016;**353**:1272–7.

Negelein E, Wulff HJ. Diphosphopyridinproteid Alkohol, acetaldehyd. *Biochem Z* 1937;**293**:351.

Nelson DL, Cox MM. *Lehninger Principles of Biochemistry*. 8th ed. New York: Macmillan, 2021.

Orla-Jensen S. Die Hauptlinien des natürlichen Bakteriensystems. *Zentralblatt Bakteriol Parasitenkd Infekt Hyg Abt II* 1909;**22**:305–46.

Pasteur L. Mémoire sur la fermentation alcoolique. *C R Acad Sci* 1857a;**45**:1032–6.

Pasteur L. Memaire sur la fermentation appelee lactique. *C R Acad Sci* 1857b;**45**:913–6.

Ragsdale SW. Pyruvate ferredoxin oxidoreductase and its radical intermediate. *Chem Rev* 2003;**103**:2333–46.

Schwann T. Vorläufige mittheilung, betreffend versuche über die weingährung und fäulniss. *Ann Phys* 1837;**117**:184–93.
